# SSR‐seq: Genotyping of microsatellites using next‐generation sequencing reveals higher level of polymorphism as compared to traditional fragment size scoring

**DOI:** 10.1002/ece3.4533

**Published:** 2018-10-25

**Authors:** Petra Šarhanová, Simon Pfanzelt, Ronny Brandt, Axel Himmelbach, Frank R. Blattner

**Affiliations:** ^1^ Leibniz Institute of Plant Genetics and Crop Plant Research (IPK) Gatersleben Germany; ^2^ Institute of Biology and Environmental Sciences Carl von Ossietzky University Oldenburg Oldenburg Germany; ^3^ Present address: Department of Botany and Biodiversity Research University of Vienna Vienna Austria; ^4^ Present address: Leibniz Institute of Plant Genetics and Crop Plant Research (IPK) Gatersleben Germany; ^5^ Present address: Max Planck Genome Centre Cologne Cologne Germany

**Keywords:** genotyping, microsatellites, multiplex PCR, next‐generation sequencing, single‐nucleotide polymorphism, size homoplasy

## Abstract

Microsatellites (or simple sequence repeats, SSR) are widely used markers in population genetics. Traditionally, genotyping was and still is carried out through recording fragment length. Now, next‐generation sequencing (NGS) makes it easy to obtain also sequence information for the loci of interest. This avoids misinterpretations that otherwise could arise due to size homoplasy. Here, an NGS strategy is described that allows to genotype hundreds of individuals at many custom‐designed SSR loci simultaneously, combining multiplex PCR, barcoding, and Illumina sequencing. We created three different datasets for which alleles were coded according to (a) length of the repetitive region, (b) total fragment length, and (c) sequence identity, in order to evaluate the eventual benefits from having sequence data at hand, not only fragment length data. For each dataset, genetic diversity statistics, as well as *F*_ST_ and *R*_ST_ values, were calculated. The number of alleles per locus, as well as observed and expected heterozygosity, was highest in the sequence identity dataset, because of single‐nucleotide polymorphisms and insertions/deletions in the flanking regions of the SSR motif. Size homoplasy was found to be very common, amounting to 44.7%–63.5% (mean over all loci) in the three study species. Thus, the information obtained by next‐generation sequencing offers a better resolution than the traditional way of SSR genotyping and allows for more accurate evolutionary interpretations.

## 1 INTRODUCTION

Microsatellites (short tandem repeats, STR, or simple sequence repeats, SSR) are widely used markers in population genetics due to their ubiquitous occurrence in the nuclear and organellar genomes, high levels of polymorphism, and codominant character. Traditionally, allele information is extracted through recording fragment length, which serves as a proxy for the number of repetitive units and is used to calculate genetic and evolutionary distance between individuals. Nonetheless, single‐nucleotide polymorphisms (SNPs) or insertions/deletions (indel) polymorphisms in the nucleotide sequence of that fragment, either within the repetitive array or in the flanking regions (FR), remain undetected by length assessment alone. Moreover, indels in the flanking regions might be incorrectly confounded with size mutations of the SSR. Thus, using only length information, SSR alleles may appear identical in state (i.e., length/size), but actually they are not necessarily identical by descent in case of convergent mutation(s) to the same size (“size homoplasy”, Estoup, Jarne, & Cornuet, 2002) or variability only in sequence but not in size. Estoup et al. (2002) used the term “molecularly accessible size homoplasy” to refer to the fraction of homoplasy that can be resolved by sequencing, which is only a subset of the size homoplasy that actually occurs at microsatellite loci. Still, sequencing cannot resolve homoplasy that arises from the convergence of two alleles to the same sequence and length.

As a consequence, the traditional assessment of fragment length may lead to underestimating genetic variability, inaccurate results, or even wrong evolutionary interpretations (Barthe et al., 2012; Blankenship, May, & Hedgecock, 2002; Peakall, Gilmore, Keys, Morgante, & Rafalski, 1998; summarized in Germain‐Aubrey, Nelson, Soltis, Soltis, & Gitzendanner, 2016). To overcome such errors, information about the nucleotide sequence of each allele is needed. While using NGS data from different sequencing platforms for SSR marker development in non‐model plant species is now a common practice (Weising, Wöhrmann, & Huettel, 2015), NGS is very rarely used for SSR scoring. In order to tackle the homoplasy problem and assess FR variation, some authors combined cloning or single‐strand conformation polymorphism and sequencing (e.g., Germain‐Aubrey et al., 2016; Lia, Bracco, Gottlieb, Poggio, & Confalonieri, 2007; Ortí, Pearse, & Avise, 1997; Šarhanová et al., 2017), but these methods are costly, time‐consuming, and not easily applicable for polyploids. There are first forays among animals (Bradbury et al., 2018; De Barba et al., 2017; Vartia et al., 2016), but comparisons between traditionally scored fragment length data and the information obtained from sequencing are still missing.

Mutations in the SSR region (predominantly changes in the number of repeats) and FR (indels and SNPs) evolve at different rates: the fast‐evolving repetitive region shows a mutation rate of about 10^−6^ to 10^−2^ per locus per generation (Schlötterer, 2000), whereas base substitutions occur at a much slower rate (depending on the genome size of the organism; Lynch, 2010), for example, in *Arabidopsis thaliana* at a rate of 7 × 10^−9^ mutations per nucleotide position per generation (Ossowski et al., 2010). The combined information from both regions can thus be used for the inference of evolutionary events at different timescales or at least indicate possible mutational saturation of the SSR region or its convergent evolution to the same size.

Here, an NGS strategy is described which allows to genotype hundreds of individuals at several, custom‐designed SSR loci simultaneously, using multiplex PCR and barcoded primers to separate individual‐specific Illumina sequence reads. Our objectives were (a) to generate nucleotide sequence data of several non‐model plant species, for which prior genomic data did not exist, from both the SSR and the flanking regions, (b) to record the length of the repetitive region, as well as SNP and indel variation within the SSR and the FR, (3) to estimate the amount of molecularly accessible size homoplasy of each locus, and (4) to compare the degree of genetic variability between different datasets based on the number of repeat units, fragment length, and sequence identity.

## 2 MATERIALS AND METHODS

### 2.1 Study species

**Figure 1:**
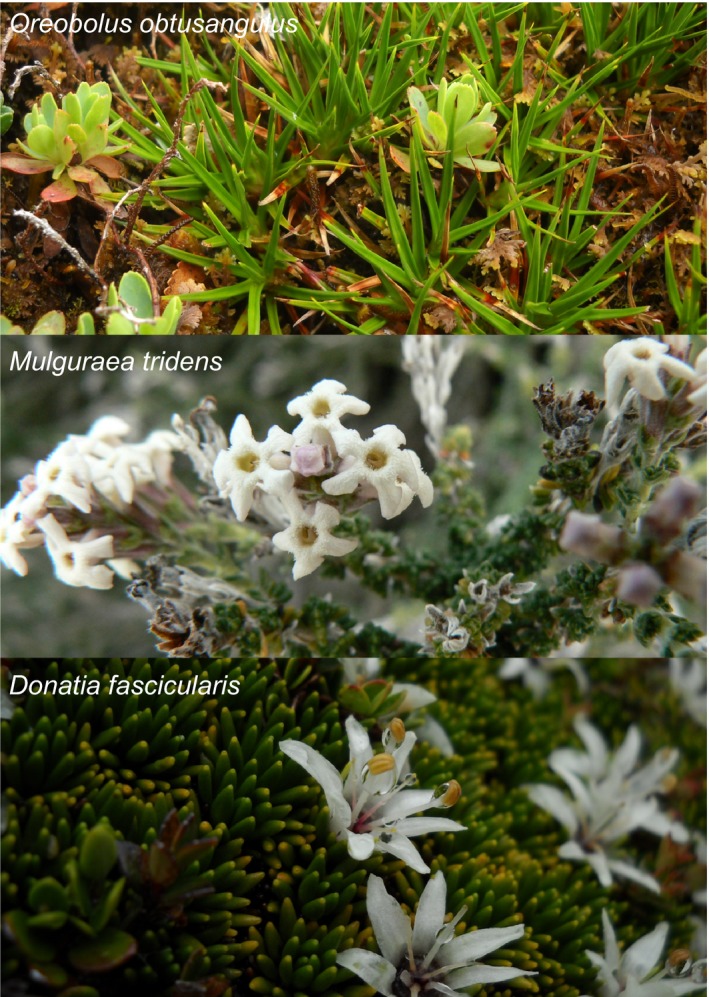
Photographs of study species

The method described here is based on three angiosperm species from southern South America: *Donatia fascicularis* (Stylidiaceae), *Mulguraea tridens* (Verbenaceae), and *Oreobolus obtusangulus* (Cyperaceae) (Table 1, Figure 1). In total, 859 individuals were genotyped at 58 nuclear SSR loci and statistically analyzed. For detailed information about two of the studied species (*D. fascicularis* and *O. obtusangulus*) and population sampling see results published elsewhere (Pfanzelt, Albach, & von Hagen, 2017; S. Pfanzelt, P. Šarhanová, D. C. Albach, & K. B. vonHagen, under review). The work is a part of a study that includes five further angiosperm species of a wide phylogenetic range, different ploidy levels, genome size, and reproductive system (*Astelia pumila*, Asteliaceae; *Berberis empetrifolia*, Berberidaceae; *Chuquiraga aurea*, Asteraceae; *Guaiacum sanctum*, Zygophyllaceae; *Rubus ulmifolius* agg., Rosaceae; in total, about 2,000 individuals were scored at 132 SSR and 3 chloroplast loci), although the data of these latter species are not included in the present study.

**Table 1:** List of study species

Species	Family, order	Ploidy level	Chromosome number	1C genome size (pg)	*N* SSR loci	*N* genotyped individuals	*N* analyzed individuals[Link]
*Donatia fascicularis* J.R.Forst. & G.Forst.	Stylidiaceae, Asterales	4×, effectively 2×	2n = 48 (Moore 1983)	3.22	20	384	328
*Mulguraea tridens* (Lag.) *N*.O'Leary & P. Peralta	Verbenaceae, Lamiales	4×	Unknown	0.86	18	88	86
*Oreobolus obtusangulus* Gaudich.	Cyperaceae, Cyperales	4×, effectively 2×	2n = 48 (Moore 1967)	0.64	20	384	360

Note

Taxonomic and genome information on the three studied species with number (*N*) of individuals and loci used. Ploidy levels of *D. fascicularis* and *O. obtusangulus* were inferred from chromosome number (4×) and from number of alleles per locus (2×); ploidy level of *M. tridens* was estimated from number of alleles per locus and through flow cytometry, using genome size as a proxy.

Individuals with missing data in more than 30% of the loci were excluded from statistical analyses.

### 2.2 SSR identification, primer design, and testing

Initial detection of SSR loci relied on assembled Illumina paired‐end sequencing reads of cDNA transcripts, which in turn stemmed from RNA extracted from fresh plant or RNA‐later (Qiagen) treated material using the RNeasy Mini Kit (Qiagen). RNA extraction followed a standard protocol and included subsequent DNA digestion and an RNA re‐precipitation step. Libraries were prepared and sequenced on an Illumina HiSeq 2000 platform according to the manufacturer's instructions, using a TruSeq RNA Library Prep Kit v2 and 10% of the lane per library. De‐novo assembly of RNA‐Seq output data was done in geneious 6.0.4 (Kearse et al., 2012) with medium sensitivity settings.

The resulting contigs from the de‐novo assembly were screened for SSRs using phobos 3.3.12 (as a plugin in geneious; Mayer, 2010). The predefined repeat unit length was 3–6 bases (to avoid frequent PCR stuttering, SSRs with dinucleotide repeats were not considered; Miller & Yuan, 1997). The minimum length of the microsatellite region was at least 21 bp. One of the primers from a given primer pair was always selected to be close to the SSR to ensure that during SSR analysis, a single NGS read contains the entire repetitive region, thus assuring correct merging of paired reads. The target length of the amplicon was up to 450 bp.

Ninety‐six primer pairs per species were designed employing primer3 (as a plugin in geneious; Rozen & Skaletsky, 2000) with the following settings: product size 250–400 bp; primer size = 18–22–30 bp (min–optimal–max); melting temperature (*T*m) = 68–70–72°C; GC content = 40%–50%–60%; maximum *T*_m_ difference = 2°C; remaining settings as default. Of these originally 96 primer pairs, 68 successfully produced amplicons for *D. fascicularis*, 51 for *M. tridens*, and 61 for *O. obtusangulus*. Amplicons of four individuals per species (different species and all loci were pooled prior library preparation generating four pools) were sequenced on an Illumina MiSeq platform (2 × 250 bp paired‐end, using MiSeq Reagent Kit v2 and 25% of a lane per library), following Meyer and Kircher (2010) and omitting fragmentation. Sequencing adapters were removed using cutadapt (Martin, 2011; minimum length 150, quality 15). Data were checked for read pairs in readtrimmchecker (Beier, 2016). Assembly was done in clc assembly cell 4.2.0 (using the clc_overlap_reads command, minimum overlap of 30), and contigs were imported into geneious. Based on intraspecific variability, up to 20 SSR loci (hereafter called target SSRs) for each species were selected.

### 2.3 Barcoding of primers and multiplex PCR

**Figure 2:**
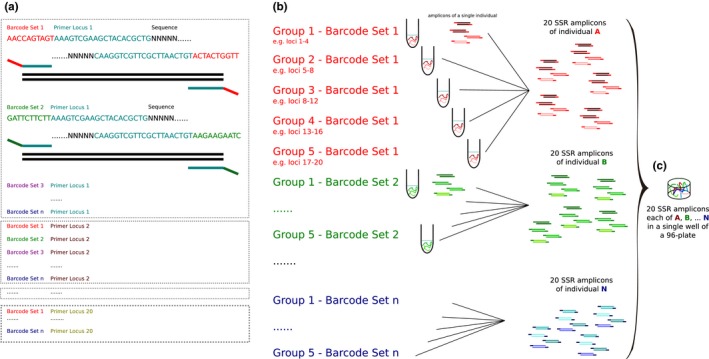
Flowchart of the laboratory procedure. (a) barcoding of primers: species A with *n* × 96 individuals genotyped at 20 loci, *n*: number of sets of barcoded primers; (b) multiplex PCRs of several loci per multiplex group (depicted here are 4 loci per multiplex group; 2–5 loci in the actual study); (c) pooling of the SSR amplicons of up to n individuals per sequencing library

DNA was extracted from silica‐dried leaf material using the DNAeasy Plant Mini Kit (Qiagen) or the innuPREP Plant DNA Kit (Analytik Jena) following the respective manufacturer´s protocol. Individuals were assigned to sample sets with 96 individuals each (four sample sets in *D. fascicularis* and *O. obtusangulus*, one in *M. tridens*). To allow for multiplexing during library construction, ten‐nucleotide barcode sequences, specific for each SSR locus and each sample set of 96 individuals, were appended to the 5ʹ‐ends of both primers (forward and reverse) of the target SSRs (Supporting Information Appendix S1). In total, 2 (forward and reverse) × 20 (loci) × number of sets (1 or 4) primers per species have been ordered. This double‐tagging allowed parallel sequencing of several conspecific samples through pooling after PCR (for a graphical description of the method see Figure 2). multiplx 2.1 (Kaplinski, Andreson, Puurand, & Remm, 2005) was used to define primer groups within each of the sample sets in order to identify optimal primer compatibility and to avoid undesired primer pairing. The grouping was done for each species and barcoded primer set separately. The software was run with the default settings, and “Calculating scores” was set to “primer‐primer any”. Each multiplex group consisted of 2–5 loci (Supporting Information Appendix S2), as multiplx 2.1 did not consider more loci to be appropriate for multiplexing due to the risk of primer dimerization.

Multiplex PCR reactions were tested on four individuals per species (amplicons of different species and all loci were pooled prior to library preparation, generating four pools) and sequenced on an Illumina MiSeq platform as described above, using 5% of a lane per library. Raw reads were processed as described in the previous step and separated by barcode (allowing single mismatches) in geneious. The numbers of reads per locus were recorded, and primer concentrations of multiplex PCRs were adjusted to achieve equal amounts (in terms of read output) of the products of each locus and multiplex reaction. The final PCR runs, with adjusted primer concentrations, were performed in 96‐well microtiter plates for each of the multiplex groups separately, using Phusion Hot Start II High‐Fidelity DNA polymerase (Thermo Fisher Scientific) or Multiplex PCR Plus Kit (Qiagen). PCR conditions are given in Supporting Information Appendix S2. The PCR products were then pooled, so that each of the 96 pools contained all target SSR loci of up to four individuals per species (individuals from different sample sets could be distinguished through the barcoded primers, Figure 2).

### 2.4 Illumina paired‐end sequencing of SSR amplicons

The 96 libraries (each including up to four individuals per species and all loci, see above) were prepared for paired‐end sequencing (2 × 250 bp) on the Illumina MiSeq platform (using the MiSeq Reagent Kit v2 and the entire flow cell), following Meyer and Kircher (2010). Because of the length of SSR amplicons (<450 bp) targeted during primer development, there was no need to perform DNA fragmentation and size selection, which reduced costs and time for library preparation.

### 2.5 Analysis pipeline

The data analysis pipeline included quality control, read merging, demultiplexing, de‐novo assembly, and the construction of reference alignments. These steps are described in detail in the following. trimgalore 0.3.7 was used for adapter clipping and pear v0.9.5 (Zhang, Kobert, Flouri, & Stamatakis, 2014) for merging of paired‐end reads (setting the p‐value threshold for the statistical test to the strictest value, i.e., 0.001) and quality trimming (quality score threshold of 30). Demultiplexing was done using the perl script fastx_barcode_splitter.pl from the fastx Toolkit. The respective barcode file contained the specific 10 bp tag and the first 10 bp of the primer sequence, so a total length of 20 bp had to be matched. Two mismatches/partial matches were allowed. Forward and reverse merged reads from the split output carrying the same tags were subsequently concatenated. De‐novo assembly was done using cap3 (version date 12/21/07; Huang & Madan, 1999), with the overlap percent identity cutoff set to 99 and the maximum gap length in any overlap set to 2. The resulting contigs (specific for each individual and each locus, for all species) were imported into geneious 6.0.4, and a multiple alignment (consensus alignment, with the threshold set to 90%) was done together with a reference sequence (original sequence from cDNA transcripts) of the respective locus and sample set barcode in order to check for mis‐tagging. Contigs were visually checked, and if variability was still present within a contig (indicating that cap3 assembled two alleles into one), de‐novo assembly was repeated in geneious (setting the maximum mismatches per read to 1%). Allele sequences (without tags and primer sequences) are deposited at NCBI GenBank (accession numbers MG322761–MG323307).

### 2.6 Size homoplasy

The amount of size homoplasy was calculated as the ratio of the number of fragment length classes containing alleles with different sequences and the total number of fragment length classes. This was done for each species and locus separately.

### 2.7 Individual error rate

Several individuals per species (seven for *D. fascicularis,* fourteen for *M. tridens*, and seven for *O. obtusangulus*) were sequenced two or more times at all loci, allowing for estimating the genotyping error rate. This was calculated based on whether identical genotypes (sequences) were observed when comparing the results of the different sequencing runs for each locus and individual.

### 2.8 Ploidy level estimation

**Figure 3:**
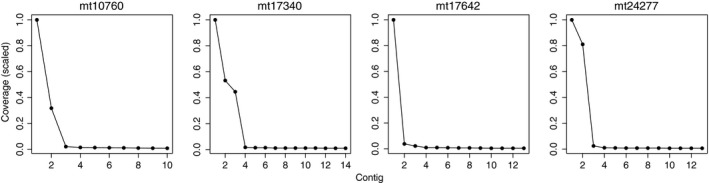
Selected coverage graphs of *Mulguraea tridens* individual Mt‐033d. Exemplarily shown are the four loci mt10760, mt17340, mt17642, and mt24277. Read coverage of contigs is scaled to 1. Contigs are numbered according to read coverage, that is, the contig with the most reads is numbered contig 1. Heterozygosity and allele dosage become apparent when comparing relative read numbers: Individual Mt‐033d is heterozygous at loci mt10760 (allele dosage 3:1), mt17340 (2:1:1), and mt24277 (2:2) and homozygous at locus mt17642

The individuals of *D. fasciularis* and *O. obtusangulus* had maximally two alleles per locus and were scored as effective diploids, although chromosome numbers point to tetraploidy. By contrast, *M. tridens* had up to four alleles per locus and individual, and the allele dosage for each individual could be determined based on read coverage ratios of the contigs (Figure 3). Therefore, each *M. tridens* individual was scored as tetraploid, with four alleles: (a) homozygous; (b) heterozygous with variable allele dosages (3:1, 2:1:1, 1:1:1:1, or 2:2). The ploidy level of *M. tridens* was also estimated through flow cytometry, using genome size as a proxy (data not provided).

### 2.9 Statistical analyses of population genetic structure and diversity

Data analyses were based on three different datasets, for which alleles were coded according to (a) length of the repetitive region (SSR‐length dataset), (b) total fragment length (fragment‐length dataset), and (c) sequence identity (sequence‐identity dataset).

spagedi 1.5a (Hardy & Vekemans, 2002) was used to calculate the total number of alleles *N*_A_, gene diversity *H*_e_ (corrected for sample size, Nei, 1978), and observed heterozygosity *H*_o_, as well as global *F*‐ and R‐statistics for each of the three datasets separately (see above). To estimate the effects of the infinite allele (IAM; Kimura & Crow, 1964) versus stepwise mutation (SMM; Ohta & Kimura, 1973) models, we compared *F*_ST_ versus *R*_ST_ of the SSR‐length and fragment‐length datasets and tested whether observed *R*_ST_ was significantly higher than its value after permutation. P‐values were obtained after 10,000 permutations of allele sizes among alleles within loci to test the null hypothesis that stepwise mutations do not contribute to genetic differentiation (Hardy, Charbonnel, Fréville, & Heuertz, 2003).

Additionally, we scored the number of variable sites, that is, SNPs and indels in the flanking and the repetitive regions plus the variation of the number of SSR units (if variable). Pearson coefficients were calculated to detect correlations between mean fragment length and the number of variable sites, number of SNPs, and degree of size homoplasy per locus; and between degree of size homoplasy and number of variable sites and SNPs. Paired Student's *t* test was used to test whether *H*_e_ and *H*_o_ differed significantly between the fragment‐length and the sequence‐identity datasets.

## 3 RESULTS

### 3.1 Output statistics

Total number of raw reads from the Illumina MiSeq run was 41,990,310 (containing all eight species). Of these, 97.7% could be unambiguously assigned to the respective libraries based on sequencing adapters. Raw read numbers per library averaged 213,594 ± 57,455 (mean ± *SD*; forward and reverse libraries yet unmerged). pear successfully merged 99.6% of all read pairs passing quality control (mean over all 96 libraries), so that the total number of merged reads was 20,401,853 (i.e., 2.8% of the raw read output either did not pass quality control or lacked its respective mate).

With regard to the three species studied here, all loci could be recovered by demultiplexing, but the average number of reads per locus (within one species) differed among loci by up to three orders of magnitude (read coverage threshold ≥10). Five and seven loci of *D. fascicularis* and *O. obtusangulus* had low coverage (<10 reads per allele) in more than 10% of the individuals or contained putative null alleles and were excluded from all analyses. One locus of *D. fascicularis* and one of *M. tridens* contained highly divergent allele sequences, suggesting the existence of two or more paralogous copies. These loci were also excluded. Locus mt11151 of *M. tridens* contained two different repetitive regions and was separated into two loci in the SSR‐length dataset. Individuals with missing data in more than 30% of the loci were excluded from the statistical analyses (Table 1).

All assemblies produced contigs with skewed read coverage distributions, that is, those contigs that represented the “true alleles” had much higher average coverage than the remaining contigs (“noise”; see Figure 3). Noise was caused by PCR recombinants (Meyerhans, Vartanian, & Wain‐Hobson, 1990; recognizable as chimera of the most common alleles) and sequencing errors (SNPs with <1% occurrence among all reads of the specific allele and individual).

Several individuals per species were sequenced two or more times (during variation assessment, multiplex testing, and the final sequencing run) at all loci. This allowed for the estimation of the error rate. The same number of alleles was retrieved for each locus and individual, though sequence variation occasionally occurred. Overall error rate for *D. fascicularis* was 1.14%, 2.45% for *M. tridens*, and 1.71% for *O. obtusangulus*.

### 3.2 Size homoplasy

The sequence data revealed that size homoplasy is very common (Table 2). It differed between species (mean over all loci 44.7%–63.5%) and—to a very high degree—between loci within one species, ranging from 20% to 100% with regard to the ratio of the number of fragment size classes with sequence variability/total number of size classes. Regarding the flanking regions, SNP variation was much more common than indel variation: SNP/indel ratios were 90/11, 97/7, and 71/7 for *D. fascicularis*,* M. tridens*, and *O. obtusangulus*, respectively. Many SSR loci also contained mutated SSR motifs (Table 3).

**Table 2:** Size homoplasy of the study species per locus

Species	Locus name	Total number of fragment size classes	Fragment size classes with homoplasy	% homoplasy
*D. fascicularis*	df14769	4	2	50
*D. fascicularis*	df123709	2	2	100
*D. fascicularis*	df124143	2	1	50
*D. fascicularis*	df126453	9	2	22.2
*D. fascicularis*	df137861	4	2	50
*D. fascicularis*	df138027	4	3	75
*D. fascicularis*	df142807	2	2	100
*D. fascicularis*	df22716	4	2	50
*D. fascicularis*	df51291	9	2	22.2
*D. fascicularis*	df61486	4	1	25
*D. fascicularis*	df79494	13	5	38.5
*D. fascicularis*	df80221	6	4	66.7
*D. fascicularis*	df80820	3	3	100
*D. fascicularis*	df91667	10	3	30
Total		76	34	44.7
*M. tridens*	mt10760	3	2	66.7
*M. tridens*	mt11151	13	4	30.8
*M. tridens*	mt14700	4	3	75
*M. tridens*	mt16240	6	3	50
*M. tridens*	mt16881	2	1	50
*M. tridens*	mt17340	4	1	25
*M. tridens*	mt17642	4	2	50
*M. tridens*	mt21753	5	1	20
*M. tridens*	mt23026	2	1	50
*M. tridens*	mt24277	4	1	25
*M. tridens*	mt25107	6	2	33.3
*M. tridens*	mt25266	7	5	71.4
*M. tridens*	mt27365	7	3	42.9
*M. tridens*	mt28267	1	1	100
*M. tridens*	mt30890	2	1	50
*M. tridens*	mt34724	1	1	100
*M. tridens*	mt57863	3	2	66.7
Total		74	34	45.9
*O. obtusangulus*	oo12746	5	2	40
*O. obtusangulus*	oo14265	3	3	100
*O. obtusangulus*	oo16914	3	2	66.7
*O. obtusangulus*	oo17752	3	2	66.7
*O. obtusangulus*	oo20129	4	2	50
*O. obtusangulus*	oo20553	3	2	66.7
*O. obtusangulus*	oo25879	4	2	50
*O. obtusangulus*	oo34170	4	3	75
*O. obtusangulus*	oo40886	8	4	50
*O. obtusangulus*	oo41307	8	7	87.5
*O. obtusangulus*	oo48962	10	5	50
*O. obtusangulus*	oo56658	5	3	60
*O. obtusangulus*	oo59128	3	3	100
Total		63	40	63.5

Note

Homoplasy is defined as the number of fragment size classes containing hidden variation (i.e., alleles of the same length but differing in sequence) divided by the total number of fragment size classes. Per‐locus percentages and the mean over all loci are given. Locus names carrying the prefix df correspond to *Donatia fascicularis*, whereas mt and oo stand for *Mulguraea tridens* and *Oreobolus obtusangulus*.

**Table 3:** Information on the used SSR loci

Locus name	SSR motif	Mean SSR length (bp)	Mutated SSR motif	Fragment length (bp)	Mean fragment length (bp)	*N* variable sites	*N* SNPs	*N* indels	Indel length (bp)
df14769	(CAC)_5__−__7, 9, 10_	22.2	Yes	367–382	374.5	6	5	0	0
df123709	(AGG)_6, 7_	19.5	No	329–344	336.5	9	8	0	0
df124143	(TGG)_4, 7, 9_	20	No	369–384	375.5	12	11	0	0
df126453	(CTT)_4, 7__−__10, 14_	27.7	No	315–345	330	4	3	0	0
df137861	(ACA)_6__−__8_	21	Yes	318–324	321	10	8	1	2
df138027	(TTCTGA)_1, 3__−__5_	18	No	289–313	301	6	5	0	0
df142807	(CTTTGC)_3_	18	No	288–301	294.5	9	8	1	13
df22716	(CAAAC)_2__−__5_	14	No	329–344	336.5	6	5	0	0
df51291	(CTT)_4, 6, 7, 9__−__13_	27.75	Yes	318–345	331.5	11	8	2	1, 8
df61486	(CAAGG)_4__−__6_	25	No	289–299	294	6	4	1	1
df79494	(CTCA)_2, 4, 7, 9__−__13, 15_	36.7	Yes	253–305	279	4	2	1	9
df80221	(GGT)_4__−__9_	19.5	No	314–329	321.5	8	7	0	0
df80820	(TTG)_6, 8, 9_	23	No	304–314	309	10	8	1	1
df91667	(ATAG)_3, 5__−__10_	26.9	No	243–266	254.5	13	8	4	2, 2, 7, 15
					Total	114	90	11	
mt10760	(AAG)_6__−__9_	22.5	No	337–346	341	2	1	0	0
mt11151^a^	(TGA)_3, 6__−__8, 10__−__13_/(TCC)_4__−__6_	26.25/15	Yes	330–363	350.5	19	18	1	3
mt14700	(GAT)_6, 7, 10_	23	No	341–353	347	6	4	1	3
mt16240	(GAA)_6__−__11_	25.5	Yes	301–316	308.5	7	6	0	0
mt16881	(ATA)_8, 11_	28.5	No	379, 388	383.5	5	4	0	0
mt17340	(TTGA)_3__−__6_	13.5	Yes	358–370	364	3	2	0	0
mt17642	(GAG)_6__−__9_	22.5	Yes	310–319	314.5	6	4	1	3
mt21753	(AGA)_4, 6, 7_	17	No	269–287	278.6	5	2	2	6, 15
mt23026	(CAT)_8, 10_	27	Yes	369, 375	372	7	5	1	3
mt24277	(ATC)_10, 13, 14, 17_	40.5	No	317–338	327.5	2	1	0	0
mt25107	(GAA)_5, 6, 8__−__10, 12_	25	No	286–307	296	4	3	0	0
mt25266	(ATG)_5, 10__−__14_	32.5	Yes	374–401	390.3	15	13	1	6
mt27365	(ATC)_5__−__11_	24	No	275–293	284	5	4	0	0
mt28267	(GAT)_6_	18	No	385	385	16	16	0	0
mt30890	(CTT)_6, 7_	19.5	No	210, 213	211.5	6	5	0	0
mt34724	(AAT)_6_	18	No	257	257	5	5	0	0
mt57863	(GAA)_7, 8, 10_	25	Yes	331–340	335	5	4	0	0
					Total	118	97	7	
oo12746	(CAC)_6__−__8, 10_	23.25	No	315–327	321	7	6	0	0
oo14265	(CGG)_2, 6, 9_	17	Yes	345–366	355.5	5	4	0	0
oo16914	(TGG)_7, 8, 12_	27	Yes	412–418	415	9	8	0	0
oo17752	(TTC)_7__−__8_	22.5	No	313–325	319	9	7	1	9
oo20129	(CGCCTC)_3__−__6_	27	No	326–344	335	8	6	1	12
oo20553	(GATTTG)_4__−__6_	30	Yes	356–368	362	4	3	0	0
oo25879	(AACCAA)_2__−__5_	21	Yes	334–352	343	4	3	0	0
oo34170	(GATT)_3__−__6_	18	No	307–319	313	7	5	1	4
oo40886	(TTC)_4__−__6, 9__−__12_	24.3	Yes	337–364	350.5	5	3	1	3
oo41307	(GAA)_12__−__19_	46.5	Yes	324–345	334.5	5	4	0	0
oo48962	(GAT)_7__−__16, 20_	36.8	Yes	383–410	396.5	8	6	2	3, 12
oo56658	(CAT)_10__−__14_	36	Yes	319–331	325	8	7	0	0
oo59128	(TTCTT)_4__−__6_	20	No	301–313	307	11	9	1	11
					Total	90	71	7	

Note

SSR motif: sequence of the SSR motif, number indicates how many times the motif is present. Variable sites include sequence variation in the SSR region itself and in the flanking region plus the variation in number of repetitive motifs (if the variation was present). *N* indicates numbers of variable sites, SNPs and indels, respectively. Locus names carrying the prefix df correspond to *Donatia fascicularis*, whereas mt and oo stand for *Mulguraea tridens* and *Oreobolus obtusangulus*.

a Includes two different SSR regions.

Size homoplasy was detected at all levels—among geographic populations, between different individuals of the same population, and even between alleles of the same individual (data not shown). The degree of size homoplasy was not correlated with mean fragment length, number of repetitive units, number of variable sites, or number of SNPs in the flanking regions (Pearson’s correlation coefficient <0.05, data not shown). To check for erroneous SNP calls, the number of rare alleles (occurring just once in the respective dataset) was recorded for each species and locus (Table 4). Based on the sequence‐identity dataset, the percentage of rare alleles in relation to the total number of alleles differed between species. The percentage of rare alleles was highest in tetraploid *M. tridens* (38.4%), followed by *D. fascicularis* (21%), and lowest in *O. obtusangulus* (3.5%).

**Table 4:** Total number of alleles and number of rare alleles (present only once in the respective dataset) for the three different datasets based on SSR‐length, fragment‐length (Fr length), and sequence‐identity (Seq identity)

Locus	*N* alleles			*N* rare alleles		
	SSR length	Fr length	Seq identity	SSR length	Fr length	Seq identity
df14769	4	4	13	1	1	3
df123709	2	2	11	–	–	3
df124143	2	2	6	–	–	–
df126453	9	9	11	–	–	1
df137861	3	4	12	1	1	5
df138027	4	4	8	–	–	–
df142807	1	2	8	–	–	–
df22716	4	4	8	1	1	3
df51291	8	9	13	2	3	4
df61486	3	4	6	–	–	–
df79494	12	13	20	1	2	3
df80221	6	6	17	–	–	5
df80820	3	3	9	–	–	1
df91667	7	10	15	–	1	5
Total	68	76	157	6	9	33
mt10760	4	4	5	2	2	3
mt11151a	8	13	20	1	5	10
mt11151b	3	–	–	1	–	–
mt14700	3	4	9	–	–	2
mt16240	6	6	10	1	1	5
mt16881	2	2	6	1	1	2
mt17340	4	4	6	2	2	2
mt17642	4	4	9	–	–	2
mt21753	3	5	7	–	–	1
mt23026	2	2	7	–	–	3
mt24277	4	4	5	–	–	–
mt25107	6	6	8	1	1	1
mt25266	6	7	15	2	2	9
mt27365	7	7	13	2	2	4
mt28267	1	1	13	–	–	7
mt30890	2	2	6	–	–	1
mt34724	1	1	5	–	–	1
mt57863	3	3	7	1	1	5
Total	69	75	151	14	17	58
oo12746	4	5	9	1	1	1
oo14265	3	3	6	–	–	–
oo16914	3	3	12	–	–	1
oo17752	2	3	8	–	–	–
oo20129	4	4	9	–	–	–
oo20553	3	3	10	–	–	1
oo25879	4	4	7	–	–	–
oo34170	4	4	8	–	–	–
oo40886	7	8	13	–	–	–
oo41307	8	8	23	–	–	1
oo48962	11	10	18	–	–	–
oo56658	5	5	12	–	1	1
oo59128	3	4	9	–	–	–
Total	61	64	144	1	2	5

Note

*N* indicates number of alleles. Locus names carrying the prefix df correspond to *Donatia fascicularis*, whereas mt and oo stand for *Mulguraea tridens* and *Oreobolus obtusangulus*.

### 3.3 PCR recombination

Sequencing revealed the existence of PCR recombinants in all individual assemblies. Application of non‐combinatorial barcoding enabled the detection of false alleles formed by recombination between individuals of different barcode sets. “Silent” recombinants (i.e., between individuals of the same barcode set) appeared as chimeric alleles composed of the most common “true” alleles and were detected because of lower read coverage in comparison to that of the alleles proper. The amount of recombinants within a library likely increases with increasing number of pooled individuals. Consequently, there is a trade‐off between cost and time efficiency and the amount of noise in the data, produced by recombinant DNA sequences.

### 3.4 Population genetic diversity and structure

The number of alleles *N*_A_, *H*_e_, and *H*_o_ differed between datasets (Tables 4 and 5). Whereas results were rather similar for the SSR‐length and fragment‐length datasets, genetic diversity estimates were generally higher for the sequence‐identity dataset in all three species. Expected and observed heterozygosity differed significantly (paired Student's *t* test, *p* < 0.05) between the sequence‐identity and fragment‐length datasets of *D. fascicularis*, and also *H*_e_ differed significantly (*p* < 0.01) between these datasets in the case of *O. obtusangulus*. There were no significant differences between datasets in case of *M. tridens*. For most loci, *F_ST_* values were similar between the datasets. However, for a few loci, *F*_ST_ differed markedly between the sequence‐identity dataset as compared to the SSR‐length dataset (df124143, oo20129; Table 5). Multilocus *F*_ST_ values of the sequence‐identity dataset were lower than that of the other datasets in all species, but the difference was not significant (*p* > 0.05).

**Table 5:** Genetic diversity indices for the three different datasets based on SSR‐length, fragment‐length (Fr length), and sequence‐identity (Seq identity)

	*R*_st_			*F*_st_			*H*_e_			*H*_o_		
	SSR length	Fr length	Seq identity	SSR length	Fr length	Seq identity	SSR length	Fr length	Seq identity	SSR length	Fr length	Seq identity
all loci	0.205	0.164	–	0.177	0.178	0.159	0.368	0.388	0.564	0.275	0.287	0.438
df14769	0.136	0.136	–	0.142	0.142	0.157	0.401	0.401	0.545	0.318	0.318	0.465
df123709	0.155	0.155	–	0.155	0.155	0.162	0.395	0.395	0.763	0.301	0.301	0.610
df124143	0.007	0.007	–	0.007	0.007	0.154	0.012	0.012	0.459	0.006	0.006	0.318
df126453	0.147	0.147	–	0.200	0.200	0.202	0.639	0.639	0.642	0.508	0.508	0.511
df137861	0.137	0.128	–	0.142	0.146	0.146	0.499	0.509	0.636	0.381	0.381	0.488
df138027	0.106	0.106	–	0.094	0.094	0.093	0.514	0.514	0.739	0.424	0.424	0.622
df142807	–	0.200	–	–	0.200	0.137	–	0.258	0.638	–	0.163	0.482
df22716	0.095	0.095	–	0.259	0.259	0.186	0.248	0.248	0.382	0.175	0.175	0.317
df51291	0.193	0.175	–	0.156	0.155	0.129	0.346	0.348	0.418	0.314	0.314	0.378
df61486	0.188	0.187	–	0.217	0.212	0.237	0.418	0.422	0.438	0.323	0.326	0.326
df79494	0.171	0.173	–	0.106	0.106	0.111	0.856	0.857	0.873	0.675	0.675	0.706
df80221	0.219	0.219	–	0.346	0.346	0.119	0.183	0.183	0.654	0.076	0.076	0.462
df80820	0.360	0.367	–	0.403	0.403	0.368	0.204	0.204	0.249	0.085	0.085	0.110
df91667	0.566*	0.130	–	0.230	0.234	0.216	0.431	0.437	0.455	0.259	0.262	0.338
all loci	0.111	0.095	–	0.115	0.114	0.110	0.384	0.411	0.526	0.183	0.197	0.257
mt10760	0.087	0.087	–	0.065	0.065	0.046	0.354	0.354	0.359	0.227	0.227	0.234
mt11151a	0.056	0.039	–	0.060	0.068	0.084	0.777	0.802	0.826	0.457	0.465	0.481
mt11151b	0.093	–	–	−0.002	–	–	0.089	–	–	0.027	–	–
mt14700	0.098	0.088	–	0.097	0.067	0.080	0.357	0.377	0.511	0.276	0.283	0.356
mt16240	0.219	0.219	–	0.230	0.230	0.197	0.635	0.635	0.662	0.112	0.112	0.122
mt16881	0.101	0.098	–	0.101	0.098	0.107	0.023	0.023	0.181	0.000	0.000	0.102
mt17340	0.148	0.148	–	0.217	0.217	0.184	0.497	0.497	0.657	0.260	0.260	0.395
mt17642	0.171	0.171	–	0.127	0.114	0.121	0.261	0.271	0.458	0.185	0.193	0.24
mt21753	0.156	0.080	–	0.160	0.147	0.120	0.530	0.557	0.620	0.269	0.302	0.339
mt23026	0.008	0.008	–	0.008	0.008	0.007	0.435	0.435	0.637	0.261	0.261	0.362
mt24277	0.185	0.18 5	–	0.157	0.157	0.198	0.470	0.470	0.502	0.154	0.154	0.162
mt25107	0.041	0.041	–	0.087	0.087	0.127	0.508	0.508	0.562	0.220	0.220	0.22
mt25266	0.163	0.117	–	0.097	0.096	0.078	0.642	0.729	0.757	0.226	0.244	0.302
mt27365	0.095	0.095	–	0.086	0.086	0.105	0.786	0.786	0.826	0.485	0.485	0.517
mt28267	–	–	–	–	–	0.035	0.000	0.000	0.489	0.000	0.000	0.24
mt30890	0.116	0.116	–	0.116	0.116	0.114	0.502	0.502	0.658	0.128	0.128	0.156
mt34724	–	–	–	–	–	0.266	0.000	0.000	0.170	0.000	0.000	0.091
mt57863	−0.052	−0.052	–	0.062	0.062	0.016	0.038	0.038	0.071	0.013	0.013	0.043
all loci	0.790***	0.785***	–	0.683	0.668	0.633	0.513	0.523	0.682	0.018	0.019	0.026
oo12746	0.803	0.734	–	0.887	0.784	0.740	0.401	0.574	0.603	0.003	0.006	0.006
oo14265	0.853	0.853	–	0.773	0.773	0.752	0.547	0.547	0.636	0.014	0.014	0.002
oo16914	0.632	0.593	–	0.663	0.599	0.642	0.512	0.396	0.689	0.003	0.006	0.008
oo17752	0.893	0.800	–	0.893	0.881	0.785	0.410	0.414	0.633	–	–	0.003
oo20129	0.191	0.513	–	0.121	0.466	0.447	0.081	0.152	0.603	–	–	0.042
oo20553	0.687	0.687	–	0.711	0.711	0.693	0.493	0.493	0.666	0.017	0.017	0.025
oo25879	0.834	0.834	–	0.814	0.814	0.760	0.585	0.585	0.629	0.017	0.017	0.019
oo34170	0.852	0.850	–	0.859	0.815	0.519	0.422	0.579	0.794	0.014	0.017	0.031
oo40886	0.851*	0.856*	–	0.741	0.709	0.692	0.637	0.642	0.649	0.014	0.014	0.014
oo41307	0.422	0.422	–	0.320	0.320	0.375	0.742	0.742	0.860	0.086	0.086	0.089
oo48962	0.725	0.489	–	0.597	0.501	0.600	0.761	0.600	0.775	0.019	0.019	0.019
oo56658	0.494	0.508	–	0.580	0.580	0.679	0.453	0.453	0.659	0.019	0.019	0.025
oo59128	0.794	0.829	–	0.707	0.703	0.662	0.621	0.623	0.674	0.028	0.028	0.031

Note

*H*_e_: gene diversity corrected for sample size. *H*_o_: observed heterozygosity. Locus names carrying the prefix df correspond to *Donatia fascicularis*, whereas mt and oo stand for *Mulguraea tridens* and *Oreobolus obtusangulus*.

*, **, and *** denote significance at *α* = 0.05, 0.01, and 0.001, respectively.

Mean permuted *R*_ST_ values were significantly lower than the observed values in some instances in *D. fascicularis* (df91667, SSR‐length dataset) and *O. obtusangulus* (oo40886 and over all loci, both SSR‐ and fragment‐length datasets). In *M. tridens*, there was no difference between observed and mean permuted *R*_ST_ values at any locus.

The higher resolution of the sequence‐identity dataset became apparent in the higher number of alleles per locus for all loci and was itself highly variable between loci, that is, the number of alleles, as calculated from the sequence dataset, was 1.2–8.0, 1.3–13, and 1.6–4.0 times higher in *D. fascicularis*,* M. tridens*, and *O. obtusangulus*, respectively, than the number of alleles calculated from the SSR‐length dataset (Table 4). In case of *M. tridens*, two loci (mt28267 and mt34724) had no variation in length of neither the SSR nor the whole fragment, but had five and thirteen alleles, respectively, based on sequence identity. There was also one locus in *D. fascicularis* (df142807) that was monomorphic in the SSR‐length dataset, but in case of fragment‐length and sequence‐identity datasets, allele numbers were two and eight, respectively.

## 4 DISCUSSION

Using Mark Twain’s “the report of my death was an exaggeration” in their publication's title, Hodel et al. (2016) expressed the opinion that SSRs still represent a useful marker system because of its high mutation rates and cost‐efficiency. They reviewed different NGS methods of SSR identification and primer development and discussed the pros and cons of using genotyping‐by‐sequencing (GBS) or restriction site‐associated DNA sequencing (RAD‐seq) in comparison to SSRs. That SSRs are not dead is reflected in the development of new analytical tools, for example, for the automatic inference of SSR genotypes (Zhan et al., 2017), and in a variety of publications that employed NGS for obtaining SSR sequence data directly from PCR amplicons (Bradbury et al., 2018; De Barba et al., 2017; Vartia et al., 2016). While these studies used animals as study systems, similar approaches have not yet been applied to plants. Our method based on barcoding of PCR primers combined with multiplexed PCR reactions and Illumina sequencing enabled us to obtain sequence data of many loci and individuals in parallel. We therefore could compare the sequencing output to what we would have obtained by recording fragment length.

### 4.1 Output statistics and estimation of ploidy levels

Demultiplexing successfully recovered all loci of the three study species, although 12 out of 58 loci had low coverage (<10 reads per allele) and those were excluded from the analyses. The adjustment of relative primer concentrations within a given PCR multiplex group is rather approximate and cannot guarantee equal yield for each locus. The ploidy level of *M. tridens* has not been reported yet; however, the basic chromosome number of the genus *Mulguraea* is *x* = 10 and related species were reported to be di‐, tetra‐, and hexaploid (Botta & Brandham, 1993). Based on the number of retrieved alleles and graphs of the standardized number of reads per each allele of the respective locus and individual (Figure 3), we inferred tetraploidy for *M. tridens*. Thus, the method can be successfully applied also for tri‐ or tetraploid species. The applicability in higher polyploids has yet to be tested, but based on our data of two other species included in the original study (*Berberis microphylla*,* Chuquiraga aurea*; data not presented here) suggest that allele detection in octo‐ and higher ploids might be complicated due to the presence of PCR recombinants (Brassac & Blattner, 2015), PCR duplicates, sequencing errors, and the problem of missing single‐copy alleles.

### 4.2 Size homoplasy

All SSR loci used in our study contained SNP variation in the FR and sometimes also in the repetitive region (Table 3), that is, the true allele number per population was always higher than when only length information would have been recorded (Table 4). The mean amount of size homoplasy (see definition above) was similar between the three studied species (44.7%, 45.9%, and 63.5% for *D. fascicularis, M. tridens*, and *O. obtusangulus*, respectively; Table 2), which is surprisingly high at the species level considering that the degree of homoplasy increases with increasing time of divergence between populations and taxa (Estoup & Cornuet, 1999). On the other hand, high degrees of size homoplasy were observed in *Rubus* subgenus *Rubus* (based on cloning and sequencing of each SSR locus), detecting SNPs at all studied loci within and among the species (Šarhanová et al., 2017). Vartia et al. (2016) screened 16 individuals of Atlantic cod for homoplasy and detected that 71.7% of the analyzed loci carry sequence variation, which represented 32% of all genotypes. Unfortunately, their way of calculating homoplasy is not fully clear, so that a direct comparison with our data is not possible. Nonetheless, we expect that the amount of homoplasy will increase with an increasing number of genotyped individuals, especially if these are geographically and evolutionarily more distant to each other.

In the FR, SNPs were around 10 times more frequent than indels (Table 3). This ratio is much higher than the one reported by Mogg et al. (2002) for *Zea mays*, in which the mean ratio over all loci between SNPs and indels was 2:1. High number of SNPs could be caused by PCR/sequencing errors, although it is not very likely for several reasons: (a) Such errors would appear as rare alleles, randomly occurring in the whole dataset; (b) rare alleles would not be present in a homozygous state; (c) if present as heterozygous, the coverage of the erroneous allele would tend to be lower than the coverage of the true allele(s) of the individual. These conditions are not met for the vast majority of rare alleles, which are species, population, and/or locus specific (Table 4). Nonetheless, negligible effects of sequencing errors cannot be ruled out.

In the case of *D. fascicularis*, the lengths of the indels of the FR were not congruent with the repeat motif length of the SSR, which was opposite to *M. tridens* and *O. obtusangulus*, where six and five out of six indels, respectively, could be confounded with tri‐, tetra‐, or hexanucleotide repeats if only fragment lengths were taken into account (Table 3).

The presented method allows using longer fragments (mean locus length was 329 bp over all loci and species with a maximum length of 418 bp), as compared to the traditional way of SSR scoring, increasing the likelihood that the FR contain genetic variation. This, in fact, does not prevent short fragments of already available primers and SSR loci to be successfully genotyped applying our method (Sochor, Šarhanová, Pfanzelt, & Trávníček, 2017). Nevertheless, the degree of size homoplasy did not correlate with mean fragment length, number of variable sites or number of SNPs or SSR units. Interestingly, the shortest locus of *D. fascicularis* (df91667) had the highest number of variable sites (Table 3). In the case of eventual correlations of size homoplasy and the number of variable sites, it should be considered that the way of calculating size homoplasy in the present study does not take into account how many alleles of the same fragment size class (differing in sequence, but not in length) are present. Therefore, the detection of a further SNP variant within a given fragment size class that already contains different sequences would not lead to a higher degree of size homoplasy. Although our SSR loci originated from RNA sequencing and we obtained blast hits for some of the loci, we could not test for a possible correlation between size homoplasy and the origin of sequences, that is, whether they stem from functional genes or the noncoding portion of the genome.

Although many workers have reported on size homoplasy and the problem of hidden variation earlier (for example in plants, see Adams, Brown, & Hamilton, 2004; Barkley, Krueger, Federici, & Roose, 2009; Curtu, Finkeldey, & Gailing, 2004; Kostia, Varvio, Vakkari, & Pulkkinen, 1995; Lia et al., 2007; Peakall et al., 1998), fragment length analysis was and still is carried out without considering FR polymorphism. Taking it into account does not eliminate all homoplasy in a dataset, because back mutation to the ancestral state still can occur. Nonetheless, besides revealing SNP variation, SSR sequencing avoids genotyping errors in case of indel polymorphism, like those that were detected in six of thirteen and seventeen SSR loci in *O. obtusangulus* and *M. tridens* and in seven of fourteen in *D. fascicularis*, respectively (Table 3).

### 4.3 Estimation of genetic diversity

The statistical analyses confirmed that in all three study species, the sequence‐identity dataset conveyed more information than the SSR‐length and fragment‐length datasets (Tables 4 and 5). The overall *F*_ST_ of *O. obtusangulus* reflects its relatively high degree of intraspecific genetic differentiation, whereas the low *F*_ST_ values of *D. fascicularis* and *M. tridens* suggest no population structure in any of the datasets. Only three loci (df124143, mt34724, and oo20129) showed markedly increased *F*_ST_ values in the sequence‐identity dataset, but for most loci, *F*_ST_ values did not differ between the datasets. This implies that the additional information contained in the sequence‐identity dataset does not necessarily influence overall population genetic and diversity statistics. The output can be affected by the nature of the study system, its genetic structure, selected loci, sampling, and other variables. *Donatia fascicularis*, for instance, has a low overall *F*_ST_ and shows almost no population genetic structure (S. Pfanzelt, P. Šarhanová, D. C. Albach, & K. B. von Hagen, under review). It has only one population (with ten individuals) that is genetically distinct, but the remaining individuals belong to a single, undifferentiated cluster. In such a case, where there is no structure at all, the high resolution of the sequence‐identity dataset is not informative either. Nonetheless, if additional information can be collected by sequencing of the studied loci, it is highly recommended to do so to ensure correct evolutionary interpretations. This mirrors Peakall et al.'s (1998: 1,283) earlier, but apparently often neglected, statement: “Consequently, DNA sequencing of SSR alleles will be essential to minimize the risk of misinterpretation and to maximize the genetic information that can be obtained.” Now widely available NGS technologies make it possible to routinely score SSR alleles through sequencing.

On the other hand, genetic diversity parameters (*H*_e_ and *H*_o_) were rather similar for the SSR‐length and fragment‐length datasets, but were generally higher for the sequence‐identity dataset in all three species (Table 5). This was especially remarkable in one locus of *D. fascicularis* (df142807) and two loci of *M. tridens* (mt28267 and mt34724), which appear to be monomorphic if only fragment or SSR lengths are considered. However, these loci were highly variable based on sequence identity (8, 13, and 5 alleles, respectively; Table 4). In *D. fascicularis* and *O. obtusangulus*, there was a significant difference in *H*_e_ between the sequence‐identity dataset compared to the traditional fragment‐length dataset. This was not the case in *M. tridens*, which may simply reflect the heterozygous nature of this tetraploid species, which is visible already when analyzing the fragment size dataset.

### 4.4 Microsatellite mutation models

Permuted *R*_ST_ values suggested for all but two studied loci (df91667, oo40886; Table 5) that stepwise mutations do not significantly contribute to genetic differentiation. Interestingly, the observed *R*_ST_ value of locus df91667 was higher than the permuted *R*_ST_, indicating the fit to the SMM, but only for the SSR‐length dataset. In fact, the four indels in the FR of that locus would mask this output in case of the traditional fragment length assessment. The second locus (oo40886) shows a bimodal distribution of the number of repeats (4–6 and 9–12) and thus fits rather to the two‐phase model of microsatellite evolution (Di Rienzo et al., 1994), in which frequent single‐step, but also rare large changes in repeat number occur. The IAM does not fit to the evolution of most of the studied loci, as it does not allow for the existence of homoplasy (Estoup et al., 2002). Other models like proportional slippage/point mutation (Kruglyak, Durrett, Schug, & Aquadro, 1998), the K‐allele model (Crow & Kimura, 1970), or more complex stepwise models can better reflect the evolution of microsatellites and should be considered in future research.

## 5 SUMMARY

Our multiplex SSR sequencing strategy produced useful information about the actual nucleotide sequences of SSR amplicons and allowed for the detection and quantification of hidden variation in a large dataset of non‐model plant species. It was shown that size homoplasy is a very common phenomenon and that indel polymorphism in the FR can be erroneously confounded with length variation within the SSR region. The additional information allows for a better understanding of microsatellite mutation processes. Sequencing of SSR loci is a prospective method with the ability to detect variability on both the intra‐ and inter‐species level and thus can be suitable for both wide‐ and fine‐scale phylogeographic studies based on single marker types.

## Conflict of interest

The authors have no conflict of interests to declare.

## Author contributions

The first two authors contributed equally to this work. PS, SP, and FRB carried out the fieldwork. PS and SP performed laboratory work and data analyses. PS, AH, RB, and FRB developed the barcode approach and devised the sequencing strategy. PS, SP, and FRB wrote the manuscript. All authors contributed to and approved the final manuscript version.

## Data accessibility

Allele sequences (accession numbers MG322761‐MG323307) and RNA‐Seq raw read data (SRA accession numbers SRX4496448‐SRX4496451) were submitted to NCBI GenBank.
