# Supplementary material for: SSR‐seq: Genotyping of microsatellites using next‐generation sequencing reveals higher level of polymorphism as compared to traditional fragment size scoring
Source: Ecol Evol. 2018 Oct 25;8(22):10817–33. doi: 10.1002/ece3.4533 (PMC6262739; doi:10.1002/ece3.4533)
Supplement: Supplementary file 1 [file ECE3-8-10817-s001.docx]

Appendix Table A1. Primer sequences and tags (barcodes) of the used loci.

| locus name | primers | tags set 1 | tags set 2 | tags set 3 | tags set 4 |
| --- | --- | --- | --- | --- | --- |
| df14769 | F: GCATCTGTACCTGCCTCACCTTT | TACCAGTATG | GCGGCTACGT | AACTTAGCAG | CCTAACTCAG |
|  | R: TCCTAGCTCCCAGAAAAAGGACCT | TACCAGTATG | GCGGCTACGT | AACTTAGCAG | CCTAACTCAG |
| df123709 | F: GGATGCCAATCACCAGTGACCAT | TACTCTCGTG | CAGTAGACGT | AAGATCTGTG | CCTCTGATAG |
|  | R: ATGGAAGCTGGAGTCGCTTTCTG | TACTCTCGTG | CAGTAGACGT | AAGATCTGTG | CCTCTGATAG |
| df124143 | F: GCGATTGGGTTCTACACGGTGAA | AGCGTCGTCT | TCGATCACGT | ACCACGCACG | CGGACTAGAG |
|  | R: ACCATGAACTCAACGATTCTGAAACA | AGCGTCGTCT | TCGATCACGT | ACCACGCACG | CGGACTAGAG |
| df126453 | F: TCGCACACTCTCTCCAATGCTTC | AGTACGCTAT | TCGCACTAGT | ACCTCGTCTG | CGGTAGAGCG |
|  | R: CCATCAACAAAAGCAGCAGACCC | AGTACGCTAT | TCGCACTAGT | ACCTCGTCTG | CGGTAGAGCG |
| df137861 | F: CTTGTGACTCACACCCAGCAACT | ATAGAGTACT | GTCAGCGACT | TAGGACTGCG | TGAATGTGCG |
|  | R: CGAAGCTTCTTGAAGATGAGAGGTGA | ATAGAGTACT | GTCAGCGACT | TAGGACTGCG | TGAATGTGCG |
| df138027 | F: GGATCAACCACCTCTGTTTTGTTGA | GCTCCTACAG | TGACGTATGT | AGGAAGCGAG | TTAAAGTCAG |
|  | R: ATAACCAATTGGAGCCTCCCAGC | GCTCCTACAG | TGACGTATGT | AGGAAGCGAG | TTAAAGTCAG |
| df142807 | CCTTGACTGATGCCTCCCTTCAC | GTCTGACTAG | TGTGAGTAGT | AGGTCTGTCG | CTAATCACAG |
|  | R: CCCCATCGAAGAACCCCACTTTT | GTCTGACTAG | TGTGAGTAGT | AGGTCTGTCG | CTAATCACAG |
| df22716 | F: ATGGAAAGAGAGGGTGCTCACAC | TACTCTCGTG | AGCTCACGTA | TATACGAGTG | TTCACGTGAT |
|  | R: AATCCTATGGCGCAAAGGAGCTT | TACTCTCGTG | AGCTCACGTA | TATACGAGTG | TTCACGTGAT |
| df51291 | F: ATCATCGGATAGAGTGGTCGGCT | TCTCCGCTCG | AGTGCTACGA | ATTGGTACAG | CTTCCTGTCT |
|  | R: CCCAATTCTCTCTGTCCCTCCCT | TCTCCGCTCG | AGTGCTACGA | ATTGGTACAG | CTTCCTGTCT |
| df61486 | F: TCTGCTTCCTGACTCCGATCTGT | ACATACGCGT | CGATCGTATA | TCAGATATAG | CTTGTACTAT |
|  | R: AGTGTCCCGGACTCTCTTGGATT | ACATACGCGT | CGATCGTATA | TCAGATATAG | CTTGTACTAT |
| df79494 | F: GGCTTCCGATTCTGCATAGGCTT | GCGCGAGTAT | GCATGTACGA | CATGCACGCG | GAAGGATCGA |
|  | R: AGTTATAAACTCAAAACACTACAGGCCG | GCGCGAGTAT | GCATGTACGA | CATGCACGCG | GAAGGATCGA |
| df80221 | F: TTCGCCGGTAAAAAGTGACACCA | ACTACTATGT | CGCGTATACA | CATTGTCACG | GACCGCAGTA |
|  | R: GCAGCAGCAGCAGTAGCAAATTA | ACTACTATGT | CGCGTATACA | CATTGTCACG | GACCGCAGTA |
| df80820 | F: TGCATCAGAACTCATGAGCGCAT | ACTGTACAGT | CTACGCTCTA | CCGAGCGTCG | TTCTTACATA |
|  | R: ACCAATACAAAAGCTGTCACTATGCT | ACTGTACAGT | CTACGCTCTA | CCGAGCGTCG | TTCTTACATA |
| df91667 | F: GCGCCAAATCTGCGTGAAATACA | GGCGTATACT | GTGCAGCGTA | CCGCATACTG | GAGACAGTCA |
|  | R: TTCTCAGAACCCTCACTGCCTCT | GGCGTATACT | GTGCAGCGTA | CCGCATACTG | GAGACAGTCA |
|  |  |  |  |  |  |
|  |  |  |  |  |  |
| mt10760 | F: GTCCTCCGGCCCGTTAAAACTAA | - | - | - | - |
|  | R: TTTCCCATCACGCTTCCACTCAA | - | - | - | - |
| mt11151* | F: TCGTCCAAGCATAGGAGGGA | - | - | - | - |
|  | R: TGGACGTTTCAGCAGTTACAGA | - | - | - | - |
| mt14700 | F: AGAAGGTTTGCGTGGTCTGT | - | - | - | - |
|  | R: GTGCGGAAAGAATGAAGCTCA | - | - | - | - |
| mt16240 | F: CGCCTTCCCCATCAAAAACG | - | - | - | - |
|  | R: AAAGGAAGGAAGAGGCAGCG | - | - | - | - |
| mt16881 | F: TTGCAGGTGAATTCCACTCCTCC | - | - | - | - |
|  | R: TGCTTTTGAGCCCCCTTCTTTCA | - | - | - | - |
| mt17340 | F: ACGGAGGAGGAGGTGAAGAACAT | - | - | - | - |
|  | R: ACAACGAAATCAACAATAACTGATTCACCA | - | - | - | - |
| mt17642 | F: CTTTGGCACGATCAAGGCAG | - | - | - | - |
|  | R: GGAACCATTTTGAGGCCACC | - | - | - | - |
| mt21753 | F: TTTCTCTCCTAGAGTCTGGCGCA | - | - | - | - |
|  | R: CTGGCAGTGAGTGAGAGGTGTTG | - | - | - | - |
| mt23026 | F: ATGACCGAAACTCATTATGGCATGGA | - | - | - | - |
|  | R: AAGGAAGTGTGTCAAAAGAGCCT | - | - | - | - |
| mt24277 | F: GTCGGGCATGCTGTTGAAAA | - | - | - | - |
|  | R: CCAAATGACCCAAGAGTCGC | - | - | - | - |
| mt25107 | F: AGAATCGGGTTCCAGTTTCCACT | - | - | - | - |
|  | R: TCCTTAGCTTTCTTGGACAGCCA | - | - | - | - |
| mt25266 | F: GCATGGTTCTTCAAAGCCCG | - | - | - | - |
|  | R: ATCAAGACAAGGTGTGGCCA | - | - | - | - |
| mt27365 | F: TCTGGCATGTACCTGTTTGACA | - | - | - | - |
|  | R: CGTCGTGAAGGTTTTCGAGG | - | - | - | - |
| mt28267 | F: CGTGCGAGAGGTTCAGAACT | - | - | - | - |
|  | R: CACCGAAACTGAGATCCCGT | - | - | - | - |
| mt30890 | F: ATCCCTTAGCCTCATGCAGATCG | - | - | - | - |
|  | R: AAAAGGCGTACCGTACTTTAACGA | - | - | - | - |
| mt34724 | F: TGTAACCCAATCGAAGCTGCAGG | - | - | - | - |
|  | R: TCAGGAGGAGTTGGCCAAGAAAT | - | - | - | - |
| mt57863 | F: AAGGGGATTAGGGATGGGACTCA | - | - | - | - |
|  | R: TCCACCGCCTCTGGAGTAAGATT | - | - | - | - |
|  |  |  |  |  |  |
|  |  |  |  |  |  |
| oo12746 | F: GGAATCGCTAGCTATACCTCGCA | AATATCATCA | ATGGACTAGC | TTACTCTATC | GAGTCGAGTG |
|  | R: GCTCCTGCTCACTCGGTTTCTAC | AATATCATCA | ATGGACTAGC | TTACTCTATC | GAGTCGAGTG |
| oo14265 | F: CCACCACTCTCAAGCCCCAAATT | ACACCGCATA | AATCGTGATC | TTGAGACATC | GATACGCTCG |
|  | R: GAGTGGTGTAGGTGATGGCCAAG | ACACCGCATA | AATCGTGATC | TTGAGACATC | GATACGCTCG |
| oo16914 | F: GCGGGAAGATCGTCGGATATGTT | ATGATAGTGA | ATATAGTCGC | TGGTGCTCTC | GCTGTATCGC |
|  | R: ACAGCATGCTTGTGATCAGCAGA | ATGATAGTGA | ATATAGTCGC | TGGTGCTCTC | GCTGTATCGC |
| oo17752 | F: CTTCACTGCCTGTGCTGTCTGAT | ATGTTGCGTA | TCTTACTGAC | TATTAGACAC | GCTTGTGTCT |
|  | R: CCGCCTCCTTTTCTCGGTCATAC | ATGTTGCGTA | TCTTACTGAC | TATTAGACAC | GCTTGTGTCT |
| oo20129 | F: ACTCAACTAGTGCAAGCGCTCTC | TGAGTCAGTA | GTTGTGTCGC | CCAATGACGC | GGACCTCTAT |
|  | R: GGTTTCCCTCGTTCGAATCACCA | TGAGTCAGTA | GTTGTGTCGC | CCAATGACGC | GGACCTCTAT |
| oo20553 | F: GCTTCTAGATGCAGGAGCTCCAC | ATGCGTGTGA | CATCCTCTAC | CCAGGTGTAC | GGAGGATACG |
|  | R: GGTGTCGAGACTAGGGTTAGGGT | ATGCGTGTGA | CATCCTCTAC | CCAGGTGTAC | GGAGGATACG |
| oo25879 | F: CAATTCCTTCCCGCAAGCACATG | CTGCGTCGCA | CTAGACGCGC | ACGGCGCTAC | GGCTGAGAGA |
|  | R: GAGCAGCATCATCCCAAAGGTCA | CTGCGTCGCA | CTAGACGCGC | ACGGCGCTAC | GGCTGAGAGA |
| oo34170 | F: TCTCTGCCATTCGTTCGTTCGTT | ACACATACGC | CGCTTGCGAC | AGAAGCAGCG | GGTATACGAC |
|  | R: CACCGCATTAAATCATCACCCGC | ACACATACGC | CGCTTGCGAC | AGAAGCAGCG | GGTATACGAC |
| oo40886 | F: AGCTTTTCATCAACTCGTGCACT | CTGATCGTGC | CGTTGATCTC | AGCCACAGAG | GGTCCACGTA |
|  | R: CAAGCGGCTTAGCGATCTCTTCT | CTGATCGTGC | CGTTGATCTC | AGCCACAGAG | GGTCCACGTA |
| oo41307 | F: GCCGGCTCTCTCAGAACAATCAA | TGCACAGCTC | GACGTCACTC | CAGCAGTGTG | GGTTCTACGT |
|  | R: TCAGCCTCAAAGTTGTTGGAGCA | TGCACAGCTC | GACGTCACTC | CAGCAGTGTG | GGTTCTACGT |
| oo48962 | F: ACCCAGTGATGGATGTGAGGTCT | TGCGATCTAC | CTGTACATAC | CTCGTGACTG | GTCGCGACTA |
|  | R: AAACAACTGGCCAACGGTAAAGC | TGCGATCTAC | CTGTACATAC | CTCGTGACTG | GTCGCGACTA |
| oo56658 | F: CTTCTCTCTCCTCTCCCTTGCCA | TCTGGCGCAC | TAGCCTGCAC | GACTAGCTCG | GTGGGTAGAC |
|  | R: CGGGTCGATTTCATCACTTGGGT | TCTGGCGCAC | TAGCCTGCAC | GACTAGCTCG | GTGGGTAGAC |
| oo59128 | F: GAGGCTGCACAGGGACTTAAGTG | AGAGCGTCAC | TTGGACGCGC | GCGTCTCTCG | GTGTACGAGT |
|  | R: TGGCAACTTTGGTTTGTCCGTTG | AGAGCGTCAC | TTGGACGCGC | GCGTCTCTCG | GTGTACGAGT |

Locus names carrying the prefix df correspond to *Donatia fascicularis*, whereas mt and oo stand for *Mulguraea tridens* and *Oreobolus obtusangulus*. Primers of *M. tridens* did not carry any tag, because only 1 sample set (96 individuals) was used.
