# Supplementary material for: SSR‐seq: Genotyping of microsatellites using next‐generation sequencing reveals higher level of polymorphism as compared to traditional fragment size scoring
Source: Ecol Evol. 2018 Oct 25;8(22):10817–33. doi: 10.1002/ece3.4533 (PMC6262739; doi:10.1002/ece3.4533)
Supplement: Supplementary file 2 [file ECE3-8-10817-s002.docx]

Appendix Table A2. PCR settings and multiplex groupings.

| **Cycling instructions:** | | |  | |  |  |
| --- | --- | --- | --- | --- | --- | --- |
|  | | |  | |  |  |
| Most of the multiplex groups (see exceptions below): | | | | |  |  |
|  | | |  | |  |  |
| Initial denaturation | 98 °C | 30 s | | 1 cycle | |  |
| Denaturation | 98 °C | 10 s | |  | |  |
| Annealing | 70 °C | 20 s | | 35 cycles | |  |
| Extension | 72 °C | 20 s | |  | |  |
| Final extension | 72 °C | 10 min | | 1 cycle | |  |
|  | hold 4 °C | | |  | |  |
|  |  |  | |  | |  |
|  |  |  | |  | |  |
| *D. fascicularis* - multiplex group 5, set 2 and multiplex group 6, set 1: | | | | | |  |
|  |  |  | |  | |  |
| Initial denaturation | 95 °C | 5 min | | 1 cycle | |  |
| Denaturation | 95 °C | 30 s | |  | |  |
| Annealing | 60 °C | 90 s | | 35 cycles | |  |
| Extension | 72 °C | 90 s | |  | |  |
| Final extension | 68 °C | 10 min | | 1 cycle | |  |
|  | hold 4 °C | | |  | |  |
|  |  |  | |  | |  |
|  |  |  | |  | |  |
| *M. tridens* - multiplex groups 3, 4 and 5: |  |  | |  | |  |
|  |  |  | |  | |  |
| Initial denaturation | 98 °C | 30 s | | 1 cycle | |  |
| Denaturation | 98 °C | 10 s | |  | |  |
| Annealing | 68 °C | 20 s | | 35 cycles | |  |
| Extension | 72 °C | 20 s | |  | |  |
| Final extension | 72 °C | 10 min | | 1 cycle | |  |
|  | hold 4 °C | | |  | |  |

| **Pipetting instructions and multiplex combinations:** | |  |  |  |
| --- | --- | --- | --- | --- |
|  |  | |  |  |
| *D. fascicularis* - multiplex group 1, set 1 |  | |  |  |
| H2O | 2.08 | |  |  |
| 5x Phusion HF buffer | 1.6 | |  |  |
| dNTPs (2.5 mM) | 0.64 | |  |  |
| df29170_F (1 µM) | 0.4 | |  |  |
| df29170_R (1 µM) | 0.4 | |  |  |
| df79494_F (1 µM) | 0.24 | |  |  |
| df79494_R (1 µM) | 0.24 | |  |  |
| df124143_F (1 µM) | 0.56 | |  |  |
| df124143_R (1 µM) | 0.56 | |  |  |
| Phusion Hot Start II DNA Polymerase (2 U/µL) | 0.08 | |  |  |
| DNA (10 ng/µL) | 1.2 | |  |  |
|  |  | |  |  |
|  |  | |  |  |
|  |  | |  |  |
| *D. fascicularis* - multiplex group 2, set 1 |  | |  |  |
| H2O | 1.76 | |  |  |
| 5x Phusion HF buffer | 1.6 | |  |  |
| dNTPs (2.5 mM) | 0.64 | |  |  |
| df22716_F (1 µM) | 0.32 | |  |  |
| df22716_R (1 µM) | 0.32 | |  |  |
| df89905_F (1 µM) | 0.64 | |  |  |
| df89905_R (1 µM) | 64 | |  |  |
| df123709_F (1 µM) | 0.4 | |  |  |
| df123709_R (1 µM) | 0.4 | |  |  |
| Phusion Hot Start II DNA Polymerase (2 U/µL) | 0.08 | |  |  |
| DNA (10 ng/µL) | 1.2 | |  |  |
|  |  | |  |  |
|  |  | |  |  |
|  |  | |  |  |
| *D. fascicularis* - multiplex group 3, set 1 |  | |  |  |
| H2O | 1.28 | |  |  |
| 5x Phusion HF buffer | 1.6 | |  |  |
| dNTPs (2.5 mM) | 0.64 | |  |  |
| df14769_F (1 µM) | 0.4 | |  |  |
| df14769_R (1 µM) | 0.4 | |  |  |
| df45878_F (1 µM) | 0.4 | |  |  |
| df45878_R (1 µM) | 0.4 | |  |  |
| df80820_F (1 µM) | 0.4 | |  |  |
| df80820_R (1 µM) | 0.4 | |  |  |
| df91667_R (1 µM) | 0.4 | |  |  |
| df91667_R (1 µM) | 0.4 | |  |  |
| Phusion Hot Start II DNA Polymerase (2 U/µL) | 0.08 | |  |  |
| DNA (10 ng/µL) | 1.2 | |  |  |
|  |  | |  |  |
|  |  | |  |  |
|  |  | |  |  |
| *D. fascicularis* - multiplex group 4, set 1 |  | |  |  |
| H2O | 1.28 | |  |  |
| 5x Phusion HF buffer | 1.6 | |  |  |
| dNTPs (2.5 mM) | 0.64 | |  |  |
| df45361_F (1 µM) | 0.56 | |  |  |
| df45361_R (1 µM) | 0.56 | |  |  |
| df61486_F (1 µM) | 0.24 | |  |  |
| df61486_R (1 µM) | 0.24 | |  |  |
| df94441_F (1 µM) | 0.24 | |  |  |
| df94441_R (1 µM) | 0.24 | |  |  |
| df126453_R (1 µM) | 0.64 | |  |  |
| df126453_R (1 µM) | 0.64 | |  |  |
| Phusion Hot Start II DNA Polymerase (2 U/µL) | 0.08 | |  |  |
| DNA (10 ng/µL) | 1.2 | |  |  |
|  |  | |  |  |
|  |  | |  |  |
|  |  | |  |  |
| *D. fascicularis* - multiplex group 5, set 1 |  | |  |  |
| H2O | 1.92 | |  |  |
| 5x Phusion HF buffer | 1.6 | |  |  |
| dNTPs (2.5 mM) | 0.64 | |  |  |
| df51291_F (1 µM) | 0.56 | |  |  |
| df51291_R (1 µM) | 0.56 | |  |  |
| df138027_F (1 µM) | 0.16 | |  |  |
| df138027_R (1 µM) | 0.16 | |  |  |
| df174177_F (1 µM) | 0.56 | |  |  |
| df174177_R (1 µM) | 0.56 | |  |  |
| Phusion Hot Start II DNA Polymerase (2 U/µL) | 0.08 | |  |  |
| DNA (10 ng/µL) | 1.2 | |  |  |
|  |  | |  |  |
|  |  | |  |  |
|  |  | |  |  |
| *D. fascicularis* - multiplex group 6, set 1 |  | |  |  |
| H2O | 1.84 | |  |  |
| Qiagen Multiplex PCR Plus Mastermix | 4 | |  |  |
| df80221_F (1 µM) | 0.16 | |  |  |
| df80221_R (1 µM) | 0.16 | |  |  |
| df137861_F (1 µM) | 0.16 | |  |  |
| df137861_R (1 µM) | 0.16 | |  |  |
| df142807_F (1 µM) | 0.16 | |  |  |
| df142807_R (1 µM) | 0.16 | |  |  |
| DNA (10 ng/µL) | 1.2 | |  |  |
|  |  | |  |  |
|  |  | |  |  |
|  |  | |  |  |
| *D. fascicularis* - multiplex group 1, set 2 |  | |  |  |
| H2O | 1.28 | |  |  |
| 5x Phusion HF buffer | 1.6 | |  |  |
| dNTPs (2.5 mM) | 0.64 | |  |  |
| df45878_F (1 µM) | 0.24 | |  |  |
| df45878_R (1 µM) | 0.24 | |  |  |
| df80820_F (1 µM) | 0.56 | |  |  |
| df80820_R (1 µM) | 0.56 | |  |  |
| df123709_F (1 µM) | 0.16 | |  |  |
| df123709_R (1 µM) | 0.16 | |  |  |
| df138027_F (1 µM) | 0.32 | |  |  |
| df138027_R (1 µM) | 0.32 | |  |  |
| df174177_F (1 µM) | 0.32 | |  |  |
| df174177_R (1 µM) | 0.32 | |  |  |
| Phusion Hot Start II DNA Polymerase (2 U/µL) | 0.08 | |  |  |
| DNA (10 ng/µL) | 1.2 | |  |  |
|  |  | |  |  |
|  |  | |  |  |
|  |  | |  |  |
| *D. fascicularis* - multiplex group 2, set 2 |  | |  |  |
| H2O | 0.96 | |  |  |
| 5x Phusion HF buffer | 1.6 | |  |  |
| dNTPs (2.5 mM) | 0.64 | |  |  |
| df22716_F (1 µM) | 0.32 | |  |  |
| df22716_R (1 µM) | 0.32 | |  |  |
| df80221_F (1 µM) | 0.48 | |  |  |
| df80221_R (1 µM) | 0.48 | |  |  |
| df94441_F (1 µM) | 0.4 | |  |  |
| df94441_R (1 µM) | 0.4 | |  |  |
| df124143_F (1 µM) | 0.56 | |  |  |
| df124143_R (1 µM) | 0.56 | |  |  |
| Phusion Hot Start II DNA Polymerase (2 U/µL) | 0.08 | |  |  |
| DNA (10 ng/µL) | 1.2 | |  |  |
|  |  | |  |  |
|  |  | |  |  |
|  |  | |  |  |
| *D. fascicularis* - multiplex group 3, set 2 |  | |  |  |
| H2O | 2.08 | |  |  |
| 5x Phusion HF buffer | 1.6 | |  |  |
| dNTPs (2.5 mM) | 0.64 | |  |  |
| df51291_F (1 µM) | 0.4 | |  |  |
| df51291_R (1 µM) | 0.4 | |  |  |
| df61486_F (1 µM) | 0.24 | |  |  |
| df61486_R (1 µM) | 0.24 | |  |  |
| df79494_F (1 µM) | 0.56 | |  |  |
| df79494_R (1 µM) | 0.56 | |  |  |
| Phusion Hot Start II DNA Polymerase (2 U/µL) | 0.08 | |  |  |
| DNA (10 ng/µL) | 1.2 | |  |  |
|  |  | |  |  |
|  |  | |  |  |
|  |  | |  |  |
| *D. fascicularis* - multiplex group 4, set 2 |  | |  |  |
| H2O | 2.08 | |  |  |
| 5x Phusion HF buffer | 1.6 | |  |  |
| dNTPs (2.5 mM) | 0.64 | |  |  |
| df45361_F (1 µM) | 0.4 | |  |  |
| df45361_R (1 µM) | 0.4 | |  |  |
| df91667_F (1 µM) | 0.56 | |  |  |
| df91667_R (1 µM) | 0.56 | |  |  |
| df126453_F (1 µM) | 0.24 | |  |  |
| df126453_R (1 µM) | 0.24 | |  |  |
| Phusion Hot Start II DNA Polymerase (2 U/µL) | 0.08 | |  |  |
| DNA (10 ng/µL) | 1.2 | |  |  |
|  |  | |  |  |
|  |  | |  |  |
|  |  | |  |  |
| *D. fascicularis* - multiplex group 5, set 2 |  | |  |  |
| Qiagen Multiplex PCR Plus Mastermix | 4 | |  |  |
| df14769_F (1 µM) | 0.35 | |  |  |
| df14769_R (1 µM) | 0.35 | |  |  |
| df29170_F (1 µM) | 0.35 | |  |  |
| df29170_R (1 µM) | 0.35 | |  |  |
| df142807_F (1 µM) | 0.35 | |  |  |
| df142807_R (1 µM) | 0.35 | |  |  |
| Phusion Hot Start II DNA Polymerase (2 U/µL) | 0.08 | |  |  |
| DNA (10 ng/µL) | 1.2 | |  |  |
|  |  | |  |  |
|  |  | |  |  |
|  |  | |  |  |
| *D. fascicularis* - multiplex group 1, set 3 |  | |  |  |
| H2O | 2.4 | |  |  |
| 5x Phusion HF buffer | 1.5 | |  |  |
| dNTPs (10 mM) | 0.15 | |  |  |
| df22761_F (1 µM) | 0.375 | |  |  |
| df22761_R (1 µM) | 0.375 | |  |  |
| df124143_F (1 µM) | 0.375 | |  |  |
| df124143_R (1 µM) | 0.375 | |  |  |
| df80221_F (1 µM) | 0.375 | |  |  |
| df80221_R (1 µM) | 0.375 | |  |  |
| Phusion Hot Start II DNA Polymerase (2 U/µL) | 0.075 | |  |  |
| DNA (10 ng/µL) | 1.125 | |  |  |
|  |  | |  |  |
|  |  | |  |  |
|  |  | |  |  |
| *D. fascicularis* - multiplex group 2, set 3 |  | |  |  |
| H2O | 1.65 | |  |  |
| 5x Phusion HF buffer | 1.5 | |  |  |
| dNTPs (10 mM) | 0.15 | |  |  |
| df45878_F (1 µM) | 0.45 | |  |  |
| df45878_R (1 µM) | 0.45 | |  |  |
| df123709_F (1 µM) | 0.3 | |  |  |
| df123709_R (1 µM) | 0.3 | |  |  |
| df126453_F (1 µM) | 0.3 | |  |  |
| df126453_R (1 µM) | 0.3 | |  |  |
| df142807_F (1 µM) | 0.45 | |  |  |
| df142807_R (1 µM) | 0.45 | |  |  |
| Phusion Hot Start II DNA Polymerase (2 U/µL) | 0.075 | |  |  |
| DNA (10 ng/µL) | 1.125 | |  |  |
|  |  | |  |  |
|  |  | |  |  |
|  |  | |  |  |
| *D. fascicularis* - multiplex group 3, set 3 |  | |  |  |
| H2O | 1.65 | |  |  |
| 5x Phusion HF buffer | 1.5 | |  |  |
| dNTPs (10 mM) | 0.15 | |  |  |
| df61486_F (1 µM) | 0.375 | |  |  |
| df61486_R (1 µM) | 0.375 | |  |  |
| df79494_F (1 µM) | 0.375 | |  |  |
| df79494_R (1 µM) | 0.375 | |  |  |
| df80820_F (1 µM) | 0.375 | |  |  |
| df80820_R (1 µM) | 0.375 | |  |  |
| df91667_F (1 µM) | 0.375 | |  |  |
| df91667_R (1 µM) | 0.375 | |  |  |
| Phusion Hot Start II DNA Polymerase (2 U/µL) | 0.075 | |  |  |
| DNA (10 ng/µL) | 1.125 | |  |  |
|  |  | |  |  |
|  |  | |  |  |
|  |  | |  |  |
| *D. fascicularis* - multiplex group 4, set 3 |  | |  |  |
| H2O | 1.65 | |  |  |
| 5x Phusion HF buffer | 1.5 | |  |  |
| dNTPs (10 mM) | 0.15 | |  |  |
| df45361_F (1 µM) | 0.0375 | |  |  |
| df45361_R (1 µM) | 0.0375 | |  |  |
| df94441_F (1 µM) | 0.0375 | |  |  |
| df94441_R (1 µM) | 0.0375 | |  |  |
| df138027_F (1 µM) | 0.0375 | |  |  |
| df138027_R (1 µM) | 0.0375 | |  |  |
| Phusion Hot Start II DNA Polymerase (2 U/µL) | 0.075 | |  |  |
| DNA (10 ng/µL) | 1.125 | |  |  |
|  |  | |  |  |
|  |  | |  |  |
|  |  | |  |  |
| *D. fascicularis* - multiplex group 5, set 3 |  | |  |  |
| H2O | 2.4 | |  |  |
| 5x Phusion HF buffer | 1.5 | |  |  |
| dNTPs (10 mM) | 0.15 | |  |  |
| df89905_F (1 µM) | 0.375 | |  |  |
| df89905_R (1 µM) | 0.375 | |  |  |
| df137861_F (1 µM) | 0.375 | |  |  |
| df137861_R (1 µM) | 0.375 | |  |  |
| df174177_F (1 µM) | 0.375 | |  |  |
| df174177_R (1 µM) | 0.375 | |  |  |
| Phusion Hot Start II DNA Polymerase (2 U/µL) | 0.075 | |  |  |
| DNA (10 ng/µL) | 1.125 | |  |  |
|  |  | |  |  |
|  |  | |  |  |
|  |  | |  |  |
| *D. fascicularis* - multiplex group 6, set 3 |  | |  |  |
| H2O | 2.4 | |  |  |
| 5x Phusion HF buffer | 1.5 | |  |  |
| dNTPs (10 mM) | 0.15 | |  |  |
| df14769_F (1 µM) | 0.375 | |  |  |
| df14769_R (1 µM) | 0.375 | |  |  |
| df29170_F (1 µM) | 0.375 | |  |  |
| df29170_R (1 µM) | 0.375 | |  |  |
| df51291_F (1 µM) | 0.375 | |  |  |
| df51291_R (1 µM) | 0.375 | |  |  |
| Phusion Hot Start II DNA Polymerase (2 U/µL) | 0.075 | |  |  |
| DNA (10 ng/µL) | 1.125 | |  |  |
|  |  | |  |  |
|  |  | |  |  |
|  |  | |  |  |
| *D. fascicularis* - multiplex group 1, set 4 |  | |  |  |
| H2O | 1.625 | |  |  |
| 5x Phusion HF buffer | 1.5 | |  |  |
| dNTPs (10 mM) | 0.15 | |  |  |
| df80221_F (1 µM) | 0.375 | |  |  |
| df80221_R (1 µM) | 0.375 | |  |  |
| df94441_F (1 µM) | 0.375 | |  |  |
| df94441_R (1 µM) | 0.375 | |  |  |
| df124143_F (1 µM) | 0.375 | |  |  |
| df124143_R (1 µM) | 0.375 | |  |  |
| df137861_F (1 µM) | 0.375 | |  |  |
| df137861_R (1 µM) | 0.375 | |  |  |
| Phusion Hot Start II DNA Polymerase (2 U/µL) | 0.075 | |  |  |
| DNA (10 ng/µL) | 1.125 | |  |  |
|  |  | |  |  |
|  |  | |  |  |
|  |  | |  |  |
| *D. fascicularis* - multiplex group 2, set 4 |  | |  |  |
| H2O | 1.625 | |  |  |
| 5x Phusion HF buffer | 1.5 | |  |  |
| dNTPs (10 mM) | 0.15 | |  |  |
| df22716_F (1 µM) | 0.375 | |  |  |
| df22716_R (1 µM) | 0.375 | |  |  |
| df45361_F (1 µM) | 0.375 | |  |  |
| df45361_R (1 µM) | 0.375 | |  |  |
| df79494_F (1 µM) | 0.375 | |  |  |
| df79494_R (1 µM) | 0.375 | |  |  |
| df142807_F (1 µM) | 0.375 | |  |  |
| df142807_R (1 µM) | 0.375 | |  |  |
| Phusion Hot Start II DNA Polymerase (2 U/µL) | 0.075 | |  |  |
| DNA (10 ng/µL) | 1.125 | |  |  |
|  |  | |  |  |
|  |  | |  |  |
|  |  | |  |  |
| *D. fascicularis* - multiplex group 3, set 4 |  | |  |  |
| H2O | 1.475 | |  |  |
| 5x Phusion HF buffer | 1.5 | |  |  |
| dNTPs (10 mM) | 0.15 | |  |  |
| df51291_F (1 µM) | 0.375 | |  |  |
| df51291_R (1 µM) | 0.375 | |  |  |
| df61486_F (1 µM) | 0.375 | |  |  |
| df61486_R (1 µM) | 0.375 | |  |  |
| df91667_F (1 µM) | 0.45 | |  |  |
| df91667_R (1 µM) | 0.45 | |  |  |
| df138027_F (1 µM) | 0.375 | |  |  |
| df138027_R (1 µM) | 0.375 | |  |  |
| Phusion Hot Start II DNA Polymerase (2 U/µL) | 0.075 | |  |  |
| DNA (10 ng/µL) | 1.125 | |  |  |
|  |  | |  |  |
|  |  | |  |  |
|  |  | |  |  |
| *D. fascicularis* - multiplex group 4, set 4 |  | |  |  |
| H2O | 1.625 | |  |  |
| 5x Phusion HF buffer | 1.5 | |  |  |
| dNTPs (10 mM) | 0.15 | |  |  |
| df45878_F (1 µM) | 0.375 | |  |  |
| df45878_R (1 µM) | 0.375 | |  |  |
| df80820_F (1 µM) | 0.375 | |  |  |
| df80820_R (1 µM) | 0.375 | |  |  |
| df89905_F (1 µM) | 0.375 | |  |  |
| df89905_R (1 µM) | 0.375 | |  |  |
| df126453_F (1 µM) | 0.375 | |  |  |
| df126453_R (1 µM) | 0.375 | |  |  |
| Phusion Hot Start II DNA Polymerase (2 U/µL) | 0.075 | |  |  |
| DNA (10 ng/µL) | 1.125 | |  |  |
|  |  | |  |  |
|  |  | |  |  |
|  |  | |  |  |
| *D. fascicularis* - multiplex group 5, set 4 |  | |  |  |
| H2O | 1.625 | |  |  |
| 5x Phusion HF buffer | 1.5 | |  |  |
| dNTPs (10 mM) | 0.15 | |  |  |
| df14769_F (1 µM) | 0.375 | |  |  |
| df14769_R (1 µM) | 0.375 | |  |  |
| df29170_F (1 µM) | 0.375 | |  |  |
| df29170_R (1 µM) | 0.375 | |  |  |
| df123709_F (1 µM) | 0.375 | |  |  |
| df123709_R (1 µM) | 0.375 | |  |  |
| df174177_F (1 µM) | 0.375 | |  |  |
| df174177_R (1 µM) | 0.375 | |  |  |
| Phusion Hot Start II DNA Polymerase (2 U/µL) | 0.075 | |  |  |
| DNA (10 ng/µL) | 1.125 | |  |  |
|  |  | |  |  |
|  |  | |  |  |
|  |  | |  |  |
| *M. tridens* - multiplex group 1 |  | |  |  |
| H2O | 3.5 | |  |  |
| 5x Phusion HF buffer | 2 | |  |  |
| dNTPs (2.5 mM) | 0.8 | |  |  |
| mt10760_F (1 µM) | 0.8 | |  |  |
| mt10760_R (1 µM) | 0.8 | |  |  |
| mt30890_F (1 µM) | 0.2 | |  |  |
| mt30890_R (1 µM) | 0.2 | |  |  |
| mt34724_F (1 µM) | 0.3 | |  |  |
| mt34724_R (1 µM) | 0.3 | |  |  |
| Phusion Hot Start II DNA Polymerase (2 U/µL) | 0.1 | |  |  |
| DNA (10 ng/µL) | 1 | |  |  |
|  |  | |  |  |
|  |  | |  |  |
|  |  | |  |  |
| *M. tridens* - multiplex group 2 |  | |  |  |
| H2O | 2.7 | |  |  |
| 5x Phusion HF buffer | 2 | |  |  |
| dNTPs (2.5 mM) | 0.8 | |  |  |
| mt17340_F | 0.7 | |  |  |
| mt17340_R | 0.7 | |  |  |
| mt23026_F | 0.4 | |  |  |
| mt23026_R | 0.4 | |  |  |
| mt25107_F | 0.6 | |  |  |
| mt25107_R | 0.6 | |  |  |
| Phusion Hot Start II DNA Polymerase (2 U/µL) | 0.1 | |  |  |
| DNA (10 ng/µL) | 1 | |  |  |
|  |  | |  |  |
|  |  | |  |  |
|  |  | |  |  |
| *M. tridens* - multiplex group 3 |  | |  |  |
| H2O | 2.7 | |  |  |
| 5x Phusion HF buffer | 2 | |  |  |
| dNTPs (2.5 mM) | 0.8 | |  |  |
| mt16881_F | 1 | |  |  |
| mt16881_R | 1 | |  |  |
| mt21753_F | 0.2 | |  |  |
| mt21753_R | 0.2 | |  |  |
| mt57863_F | 0.5 | |  |  |
| mt57863_R | 0.5 | |  |  |
| Phusion Hot Start II DNA Polymerase (2 U/µL) | 0.1 | |  |  |
| DNA (10 ng/µL) | 1 | |  |  |
|  |  | |  |  |
|  |  | |  |  |
|  |  | |  |  |
| *M. tridens* - multiplex group 4 |  | |  |  |
| H2O | 1.9 | |  |  |
| 5x Phusion HF buffer | 2 | |  |  |
| dNTPs (2.5 mM) | 0.8 | |  |  |
| mt11151_F | 0.6 | |  |  |
| mt11151_R | 0.6 | |  |  |
| mt17642_F | 0.5 | |  |  |
| mt17642_R | 0.5 | |  |  |
| mt21025_F | 0.5 | |  |  |
| mt21025_R | 0.5 | |  |  |
| mt24277_F | 0.5 | |  |  |
| mt24277_R | 0.5 | |  |  |
| Phusion Hot Start II DNA Polymerase (2 U/µL) | 0.1 | |  |  |
| DNA (10 ng/µL) | 1 | |  |  |
|  |  | |  |  |
|  |  | |  |  |
|  |  | |  |  |
| *M. tridens* - multiplex group 5 |  | |  |  |
| H2O | 0.1 | |  |  |
| 5x Phusion HF buffer | 2 | |  |  |
| dNTPs (2.5 mM) | 0.8 | |  |  |
| mt14700_F | 0.5 | |  |  |
| mt14700_R | 0.5 | |  |  |
| mt16240_F | 1 | |  |  |
| mt16240_R | 1 | |  |  |
| mt25266_F | 0.7 | |  |  |
| mt25266_R | 0.7 | |  |  |
| mt27365_F | 0.4 | |  |  |
| mt27365_R | 0.4 | |  |  |
| mt28267_F | 0.4 | |  |  |
| mt28267_R | 0.4 | |  |  |
| Phusion Hot Start II DNA Polymerase (2 U/µL) | 0.1 | |  |  |
| DNA (10 ng/µL) | 1 | |  |  |
|  |  | |  |  |
|  |  | |  |  |
|  |  | |  |  |
| *O. obtusangulus* - multiplex group 1, set 1 |  | |  |  |
| H2O | 2.72 | |  |  |
| 5x Phusion HF buffer | 1.6 | |  |  |
| dNTPs (2.5 mM) | 0.64 | |  |  |
| oo16275_F (1 µM) | 0.24 | |  |  |
| oo16275_R (1 µM) | 0.24 | |  |  |
| oo16914_F (1 µM) | 0.16 | |  |  |
| oo16914_R (1 µM) | 0.16 | |  |  |
| oo20553_F (1 µM) | 0.48 | |  |  |
| oo20553_R (1 µM) | 0.48 | |  |  |
| Phusion Hot Start II DNA Polymerase (2 U/µL) | 0.08 | |  |  |
| DNA (10 ng/µL) | 1.2 | |  |  |
|  |  | |  |  |
|  |  | |  |  |
|  |  | |  |  |
| *O. obtusangulus* - multiplex group 2, set 1 |  | |  |  |
| H2O | 1.6 | |  |  |
| 5x Phusion HF buffer | 1.6 | |  |  |
| dNTPs (2.5 mM) | 0.64 | |  |  |
| oo16095_F (1 µM) | 0.24 | |  |  |
| oo16095_R (1 µM) | 0.24 | |  |  |
| oo41121_F (1 µM) | 0.16 | |  |  |
| oo41121_R (1 µM) | 0.16 | |  |  |
| oo41622_F (1 µM) | 0.64 | |  |  |
| oo41622_R (1 µM) | 0.64 | |  |  |
| oo48962_F (1 µM) | 0.4 | |  |  |
| oo48962_R (1 µM) | 0.4 | |  |  |
| Phusion Hot Start II DNA Polymerase (2 U/µL) | 0.08 | |  |  |
| DNA (10 ng/µL) | 1.2 | |  |  |
|  |  | |  |  |
|  |  | |  |  |
|  |  | |  |  |
| *O. obtusangulus* - multiplex group 3, set 1 |  | |  |  |
| H2O | 2.4 | |  |  |
| 5x Phusion HF buffer | 1.6 | |  |  |
| dNTPs (2.5 mM) | 0.64 | |  |  |
| oo20000_F (1 µM) | 0.64 | |  |  |
| oo20000_R (1 µM) | 0.64 | |  |  |
| oo20699_F (1 µM) | 0.24 | |  |  |
| oo20699_R (1 µM) | 0.24 | |  |  |
| oo59128_F (1 µM) | 0.16 | |  |  |
| oo59128_R (1 µM) | 0.16 | |  |  |
| Phusion Hot Start II DNA Polymerase (2 U/µL) | 0.08 | |  |  |
| DNA (10 ng/µL) | 1.2 | |  |  |
|  |  | |  |  |
|  |  | |  |  |
|  |  | |  |  |
| *O. obtusangulus* - multiplex group 4, set 1 |  | |  |  |
| H2O | 2.56 | |  |  |
| 5x Phusion HF buffer | 1.6 | |  |  |
| dNTPs (2.5 mM) | 0.64 | |  |  |
| oo12746_F (1 µM) | 0.16 | |  |  |
| oo12746_R (1 µM) | 0.16 | |  |  |
| oo14265_F (1 µM) | 0.56 | |  |  |
| oo14265_R (1 µM) | 0.56 | |  |  |
| oo20129_F (1 µM) | 0.24 | |  |  |
| oo20129_R (1 µM) | 0.24 | |  |  |
| Phusion Hot Start II DNA Polymerase (2 U/µL) | 0.08 | |  |  |
| DNA (10 ng/µL) | 1.2 | |  |  |
|  |  | |  |  |
|  |  | |  |  |
|  |  | |  |  |
| *O. obtusangulus* - multiplex group 5, set 1 |  | |  |  |
| H2O | 1.92 | |  |  |
| 5x Phusion HF buffer | 1.6 | |  |  |
| dNTPs (2.5 mM) | 0.64 | |  |  |
| oo15422_F (1 µM) | 0.24 | |  |  |
| oo15422_R (1 µM) | 0.24 | |  |  |
| oo17752_F (1 µM) | 0.24 | |  |  |
| oo17752_R (1 µM) | 0.24 | |  |  |
| oo25879_F (1 µM) | 0.48 | |  |  |
| oo25879_R (1 µM) | 0.48 | |  |  |
| oo41307_F (1 µM) | 0.32 | |  |  |
| oo41307_R (1 µM) | 0.32 | |  |  |
| Phusion Hot Start II DNA Polymerase (2 U/µL) | 0.08 | |  |  |
| DNA (10 ng/µL) | 1.2 | |  |  |
|  |  | |  |  |
|  |  | |  |  |
|  |  | |  |  |
| *O. obtusangulus* - multiplex group 6, set 1 |  | |  |  |
| H2O | 2.56 | |  |  |
| 5x Phusion HF buffer | 1.6 | |  |  |
| dNTPs (2.5 mM) | 0.64 | |  |  |
| oo34170_F (1 µM) | 0.16 | |  |  |
| oo34170_R (1 µM) | 0.16 | |  |  |
| oo40886_F (1 µM) | 0.4 | |  |  |
| oo40886_R (1 µM) | 0.4 | |  |  |
| oo56658_F (1 µM) | 0.4 | |  |  |
| oo56658_R (1 µM) | 0.4 | |  |  |
| Phusion Hot Start II DNA Polymerase (2 U/µL) | 0.08 | |  |  |
| DNA (10 ng/µL) | 1.2 | |  |  |
|  |  | |  |  |
|  |  | |  |  |
|  |  | |  |  |
| *O. obtusangulus* - multiplex group 1, set 2 |  | |  |  |
| H2O | 2.4 | |  |  |
| 5x Phusion HF buffer | 1.5 | |  |  |
| dNTPs (10 mM) | 0.15 | |  |  |
| oo14265_F (1 µM) | 0.375 | |  |  |
| oo14265_R (1 µM) | 0.375 | |  |  |
| oo16095_F (1 µM) | 0.375 | |  |  |
| oo16095_R (1 µM) | 0.375 | |  |  |
| oo59128_F (1 µM) | 0.375 | |  |  |
| oo59128_R (1 µM) | 0.375 | |  |  |
| Phusion Hot Start II DNA Polymerase (2 U/µL) | 0.075 | |  |  |
| DNA (10 ng/µL) | 1.125 | |  |  |
|  |  | |  |  |
|  |  | |  |  |
|  |  | |  |  |
| *O. obtusangulus* - multiplex group 2, set 2 |  | |  |  |
| H2O | 2.55 | |  |  |
| 5x Phusion HF buffer | 1.5 | |  |  |
| dNTPs (10 mM) | 0.15 | |  |  |
| oo17752_F (1 µM) | 0.45 | |  |  |
| oo17752_R (1 µM) | 0.45 | |  |  |
| oo20000_F (1 µM) | 0.3 | |  |  |
| oo20000_R (1 µM) | 0.3 | |  |  |
| oo20699_F (1 µM) | 0.3 | |  |  |
| oo20699_R (1 µM) | 0.3 | |  |  |
| Phusion Hot Start II DNA Polymerase (2 U/µL) | 0.075 | |  |  |
| DNA (10 ng/µL) | 1.125 | |  |  |
|  |  | |  |  |
|  |  | |  |  |
|  |  | |  |  |
| *O. obtusangulus* - multiplex group 3, set 2 |  | |  |  |
| H2O | 2.55 | |  |  |
| 5x Phusion HF buffer | 1.5 | |  |  |
| dNTPs (10 mM) | 0.15 | |  |  |
| oo12746_F (1 µM) | 0.45 | |  |  |
| oo12746_R (1 µM) | 0.45 | |  |  |
| oo16275_F (1 µM) | 0.3 | |  |  |
| oo16275_R (1 µM) | 0.3 | |  |  |
| oo16914_F (1 µM) | 0.3 | |  |  |
| oo16914_R (1 µM) | 0.3 | |  |  |
| Phusion Hot Start II DNA Polymerase (2 U/µL) | 0.075 | |  |  |
| DNA (10 ng/µL) | 1.125 | |  |  |
|  |  | |  |  |
|  |  | |  |  |
|  |  | |  |  |
| *O. obtusangulus* - multiplex group 4, set 2 |  | |  |  |
| H2O | 2.4 | |  |  |
| 5x Phusion HF buffer | 1.5 | |  |  |
| dNTPs (10 mM) | 0.15 | |  |  |
| oo20553_F (1 µM) | 0.375 | |  |  |
| oo20553_R (1 µM) | 0.375 | |  |  |
| oo34170_F (1 µM) | 0.375 | |  |  |
| oo34170_R (1 µM) | 0.375 | |  |  |
| oo41622_F (1 µM) | 0.375 | |  |  |
| oo41622_R (1 µM) | 0.375 | |  |  |
| Phusion Hot Start II DNA Polymerase (2 U/µL) | 0.075 | |  |  |
| DNA (10 ng/µL) | 1.125 | |  |  |
|  |  | |  |  |
|  |  | |  |  |
|  |  | |  |  |
| *O. obtusangulus* - multiplex group 5, set 2 |  | |  |  |
| H2O | 2.1 | |  |  |
| 5x Phusion HF buffer | 1.5 | |  |  |
| dNTPs (10 mM) | 0.15 | |  |  |
| oo25879_F (1 µM) | 0.525 | |  |  |
| oo25879_R (1 µM) | 0.525 | |  |  |
| oo41307_F (1 µM) | 0.375 | |  |  |
| oo41307_R (1 µM) | 0.375 | |  |  |
| oo56658_F (1 µM) | 0.375 | |  |  |
| oo56658_R (1 µM) | 0.375 | |  |  |
| Phusion Hot Start II DNA Polymerase (2 U/µL) | 0.075 | |  |  |
| DNA (10 ng/µL) | 1.125 | |  |  |
|  |  | |  |  |
|  |  | |  |  |
|  |  | |  |  |
| *O. obtusangulus* - multiplex group 6, set 2 |  | |  |  |
| H2O | 2.4 | |  |  |
| 5x Phusion HF buffer | 1.5 | |  |  |
| dNTPs (10 mM) | 0.15 | |  |  |
| oo15422_F (1 µM) | 0.375 | |  |  |
| oo15422_R (1 µM) | 0.375 | |  |  |
| oo40886_F (1 µM) | 0.375 | |  |  |
| oo40886_R (1 µM) | 0.375 | |  |  |
| oo48962_F (1 µM) | 0.375 | |  |  |
| oo48962_R (1 µM) | 0.375 | |  |  |
| Phusion Hot Start II DNA Polymerase (2 U/µL) | 0.075 | |  |  |
| DNA (10 ng/µL) | 1.125 | |  |  |
|  |  | |  |  |
|  |  | |  |  |
|  |  | |  |  |
| *O. obtusangulus* - multiplex group 7, set 2 |  | |  |  |
| H2O | 3.15 | |  |  |
| 5x Phusion HF buffer | 1.5 | |  |  |
| dNTPs (10 mM) | 0.15 | |  |  |
| oo20129_F (1 µM) | 0.375 | |  |  |
| oo20129_R (1 µM) | 0.375 | |  |  |
| oo41121_F (1 µM) | 0.375 | |  |  |
| oo41121_R (1 µM) | 0.375 | |  |  |
| Phusion Hot Start II DNA Polymerase (2 U/µL) | 0.075 | |  |  |
| DNA (10 ng/µL) | 1.125 | |  |  |
|  |  | |  |  |
|  |  | |  |  |
|  |  | |  |  |
| *O. obtusangulus* - multiplex group 1, set 3 |  | |  |  |
| H2O | 1.6 | |  |  |
| 5x Phusion HF buffer | 1.6 | |  |  |
| dNTPs (2.5 mM) | 0.64 | |  |  |
| oo14265_F (1 µM) | 0.16 | |  |  |
| oo14265_R (1 µM) | 0.16 | |  |  |
| oo16914_F (1 µM) | 0.56 | |  |  |
| oo16914_R (1 µM) | 0.56 | |  |  |
| oo56658_F (1 µM) | 0.16 | |  |  |
| oo56658_R (1 µM) | 0.16 | |  |  |
| oo59128_F (1 µM) | 0.56 | |  |  |
| oo59128_R (1 µM) | 0.56 | |  |  |
| Phusion Hot Start II DNA Polymerase (2 U/µL) | 0.08 | |  |  |
| DNA (10 ng/µL) | 1.2 | |  |  |
|  |  | |  |  |
|  |  | |  |  |
|  |  | |  |  |
| *O. obtusangulus* - multiplex group 2, set 3 |  | |  |  |
| H2O | 2.4 | |  |  |
| 5x Phusion HF buffer | 1.6 | |  |  |
| dNTPs (2.5 mM) | 0.64 | |  |  |
| oo20699_F (1 µM) | 0.16 | |  |  |
| oo20699_R (1 µM) | 0.16 | |  |  |
| oo25879_F (1 µM) | 0.24 | |  |  |
| oo25879_R (1 µM) | 0.24 | |  |  |
| oo41622_F (1 µM) | 0.64 | |  |  |
| oo41622_R (1 µM) | 0.64 | |  |  |
| Phusion Hot Start II DNA Polymerase (2 U/µL) | 0.08 | |  |  |
| DNA (10 ng/µL) | 1.2 | |  |  |
|  |  | |  |  |
|  |  | |  |  |
|  |  | |  |  |
| *O. obtusangulus* - multiplex group 3, set 3 |  | |  |  |
| H2O | 2.24 | |  |  |
| 5x Phusion HF buffer | 1.6 | |  |  |
| dNTPs (2.5 mM) | 0.64 | |  |  |
| oo12746_F (1 µM) | 0.32 | |  |  |
| oo12746_R (1 µM) | 0.32 | |  |  |
| oo16275_F (1 µM) | 0.64 | |  |  |
| oo16275_R (1 µM) | 0.64 | |  |  |
| oo41307_F (1 µM) | 0.16 | |  |  |
| oo41307_R (1 µM) | 0.16 | |  |  |
| Phusion Hot Start II DNA Polymerase (2 U/µL) | 0.08 | |  |  |
| DNA (10 ng/µL) | 1.2 | |  |  |
|  |  | |  |  |
|  |  | |  |  |
|  |  | |  |  |
| *O. obtusangulus* - multiplex group 4, set 3 |  | |  |  |
| H2O | 1.76 | |  |  |
| 5x Phusion HF buffer | 1.6 | |  |  |
| dNTPs (2.5 mM) | 0.64 | |  |  |
| oo16095_F (1 µM) | 0.24 | |  |  |
| oo16095_R (1 µM) | 0.24 | |  |  |
| oo17752_F (1 µM) | 0.16 | |  |  |
| oo17752_R (1 µM) | 0.16 | |  |  |
| oo20000_F (1 µM) | 0.64 | |  |  |
| oo20000_R (1 µM) | 0.64 | |  |  |
| oo40886_F (1 µM) | 0.32 | |  |  |
| oo40886_R (1 µM) | 0.32 | |  |  |
| Phusion Hot Start II DNA Polymerase (2 U/µL) | 0.08 | |  |  |
| DNA (10 ng/µL) | 1.2 | |  |  |
|  |  | |  |  |
|  |  | |  |  |
|  |  | |  |  |
| *O. obtusangulus* - multiplex group 5, set 3 |  | |  |  |
| H2O | 3.4 | |  |  |
| 5x Phusion HF buffer | 1.6 | |  |  |
| dNTPs (2.5 mM) | 0.64 | |  |  |
| oo15422_F (1 µM) | 0.16 | |  |  |
| oo15422_R (1 µM) | 0.16 | |  |  |
| oo20129_F (1 µM) | 0.16 | |  |  |
| oo20129_R (1 µM) | 0.16 | |  |  |
| oo41121_F (1 µM) | 0.4 | |  |  |
| oo41121_R (1 µM) | 0.4 | |  |  |
| Phusion Hot Start II DNA Polymerase (2 U/µL) | 0.08 | |  |  |
| DNA (10 ng/µL) | 1.2 | |  |  |
|  |  | |  |  |
|  |  | |  |  |
|  |  | |  |  |
| *O. obtusangulus* - multiplex group 6, set 3 |  | |  |  |
| H2O | 2.24 | |  |  |
| 5x Phusion HF buffer | 1.6 | |  |  |
| dNTPs (2.5 mM) | 0.64 | |  |  |
| oo20553_F (1 µM) | 0.32 | |  |  |
| oo20553_R (1 µM) | 0.32 | |  |  |
| oo34170_F (1 µM) | 0.16 | |  |  |
| oo34170_R (1 µM) | 0.16 | |  |  |
| oo48962_F (1 µM) | 0.64 | |  |  |
| oo48962_R (1 µM) | 0.64 | |  |  |
| Phusion Hot Start II DNA Polymerase (2 U/µL) | 0.08 | |  |  |
| DNA (10 ng/µL) | 1.2 | |  |  |
|  |  | |  |  |
|  |  | |  |  |
|  |  | |  |  |
| *O. obtusangulus* - multiplex group 1, set 4 |  | |  |  |
| H2O | 2.4 | |  |  |
| 5x Phusion HF buffer | 1.5 | |  |  |
| dNTPs (10 mM) | 0.15 | |  |  |
| oo15422_F (1 µM) | 0.375 | |  |  |
| oo15422_R (1 µM) | 0.375 | |  |  |
| oo25879_F (1 µM) | 0.375 | |  |  |
| oo25879_R (1 µM) | 0.375 | |  |  |
| oo48962_F (1 µM) | 0.375 | |  |  |
| oo48962_R (1 µM) | 0.375 | |  |  |
| Phusion Hot Start II DNA Polymerase (2 U/µL) | 0.075 | |  |  |
| DNA (10 ng/µL) | 1.125 | |  |  |
|  |  | |  |  |
|  |  | |  |  |
|  |  | |  |  |
| *O. obtusangulus* - multiplex group 2, set 4 |  | |  |  |
| H2O | 2.4 | |  |  |
| 5x Phusion HF buffer | 1.5 | |  |  |
| dNTPs (10 mM) | 0.15 | |  |  |
| oo12746_F (1 µM) | 0.375 | |  |  |
| oo12746_R (1 µM) | 0.375 | |  |  |
| oo16914_F (1 µM) | 0.375 | |  |  |
| oo16914_R (1 µM) | 0.375 | |  |  |
| oo20129_F (1 µM) | 0.375 | |  |  |
| oo20129_R (1 µM) | 0.375 | |  |  |
| Phusion Hot Start II DNA Polymerase (2 U/µL) | 0.075 | |  |  |
| DNA (10 ng/µL) | 1.125 | |  |  |
|  |  | |  |  |
|  |  | |  |  |
|  |  | |  |  |
| *O. obtusangulus* - multiplex group 3, set 4 |  | |  |  |
| H2O | 2.4 | |  |  |
| 5x Phusion HF buffer | 1.5 | |  |  |
| dNTPs (10 mM) | 0.15 | |  |  |
| oo20000_F (1 µM) | 0.375 | |  |  |
| oo20000_R (1 µM) | 0.375 | |  |  |
| oo20553_F (1 µM) | 0.375 | |  |  |
| oo20553_R (1 µM) | 0.375 | |  |  |
| oo56658_F (1 µM) | 0.375 | |  |  |
| oo56658_R (1 µM) | 0.375 | |  |  |
| Phusion Hot Start II DNA Polymerase (2 U/µL) | 0.075 | |  |  |
| DNA (10 ng/µL) | 1.125 | |  |  |
|  |  | |  |  |
|  |  | |  |  |
|  |  | |  |  |
| *O. obtusangulus* - multiplex group 4, set 4 |  | |  |  |
| H2O | 2.4 | |  |  |
| 5x Phusion HF buffer | 1.5 | |  |  |
| dNTPs (10 mM) | 0.15 | |  |  |
| oo14265_F (1 µM) | 0.375 | |  |  |
| oo14265_R (1 µM) | 0.375 | |  |  |
| oo17752_F (1 µM) | 0.375 | |  |  |
| oo17752_R (1 µM) | 0.375 | |  |  |
| oo41622_F (1 µM) | 0.375 | |  |  |
| oo41622_R (1 µM) | 0.375 | |  |  |
| Phusion Hot Start II DNA Polymerase (2 U/µL) | 0.075 | |  |  |
| DNA (10 ng/µL) | 1.125 | |  |  |
|  |  | |  |  |
|  |  | |  |  |
|  |  | |  |  |
| *O. obtusangulus* - multiplex group 5, set 4 |  | |  |  |
| H2O | 2.1 | |  |  |
| 5x Phusion HF buffer | 1.5 | |  |  |
| dNTPs (10 mM) | 0.15 | |  |  |
| oo16275_F (1 µM) | 0.525 | |  |  |
| oo16275_R (1 µM) | 0.525 | |  |  |
| oo20699_F (1 µM) | 0.45 | |  |  |
| oo20699_R (1 µM) | 0.45 | |  |  |
| oo59128_F (1 µM) | 0.3 | |  |  |
| oo59128_R (1 µM) | 0.3 | |  |  |
| Phusion Hot Start II DNA Polymerase (2 U/µL) | 0.075 | |  |  |
| DNA (10 ng/µL) | 1.125 | |  |  |
|  |  | |  |  |
|  |  | |  |  |
|  |  | |  |  |
| *O. obtusangulus* - multiplex group 6, set 4 |  | |  |  |
| H2O | 2.4 | |  |  |
| 5x Phusion HF buffer | 1.5 | |  |  |
| dNTPs (10 mM) | 0.15 | |  |  |
| oo40886_F (1 µM) | 0.375 | |  |  |
| oo40886_R (1 µM) | 0.375 | |  |  |
| oo41121_F (1 µM) | 0.375 | |  |  |
| oo41121_R (1 µM) | 0.375 | |  |  |
| oo41307_F (1 µM) | 0.375 | |  |  |
| oo41307_R (1 µM) | 0.375 | |  |  |
| Phusion Hot Start II DNA Polymerase (2 U/µL) | 0.075 | |  |  |
| DNA (10 ng/µL) | 1.125 | |  |  |
|  |  | |  |  |
|  |  | |  |  |
|  |  | |  |  |
| *O. obtusangulus* - multiplex group 7, set 4 |  | |  |  |
| H2O | 3.15 | |  |  |
| 5x Phusion HF buffer | 1.5 | |  |  |
| dNTPs (10 mM) | 0.15 | |  |  |
| oo16095_F (1 µM) | 0.375 | |  |  |
| oo16095_R (1 µM) | 0.375 | |  |  |
| oo34170_F (1 µM) | 0.375 | |  |  |
| oo34170_R (1 µM) | 0.375 | |  |  |
| Phusion Hot Start II DNA Polymerase (2 U/µL) | 0.075 | |  |  |
| DNA (10 ng/µL) | 1.125 | |  |  |
|  |  | |  |  |
